# Unique Profile of Ordered Arrangements of Repetitive Elements in the C57BL/6J Mouse Genome Implicating Their Functional Roles

**DOI:** 10.1371/journal.pone.0035156

**Published:** 2012-04-18

**Authors:** Young-Kwan Lee, Kang-Hoon Lee, Seon-Gyu Kim, Ramzi Melhem, Chang-Suk Moon, Sicong Liu, David G. Greenhalgh, Kiho Cho

**Affiliations:** Department of Surgery, University of California Davis, Shriners Hospitals for Children Northern California, Sacramento, California, United States of America; University of Utah, United States of America

## Abstract

The entirety of all protein coding sequences is reported to represent a small fraction (∼2%) of the mouse and human genomes; the vast majority of the rest of the genome is presumed to be repetitive elements (REs). In this study, the C57BL/6J mouse reference genome was subjected to an unbiased RE mining to establish a whole-genome profile of RE occurrence and arrangement. The C57BL/6J mouse genome was fragmented into an initial set of 5,321 units of 0.5 Mb, and surveyed for REs using unbiased self-alignment and dot-matrix protocols. The survey revealed that individual chromosomes had unique profiles of RE arrangement structures, named RE arrays. The RE populations in certain genomic regions were arranged into various forms of complexly organized structures using combinations of direct and/or inverse repeats. Some of these RE arrays spanned stretches of over 2 Mb, which may contribute to the structural configuration of the respective genomic regions. There were substantial differences in RE density among the 21 chromosomes, with chromosome Y being the most densely populated. In addition, the RE array population in the mouse chromosomes X and Y was substantially different from those of the reference human chromosomes. Conversion of the dot-matrix data pertaining to a tandem 13-repeat structure within the Ch7.032 genome unit into a line map of known REs revealed a repeat unit of ∼11.3 Kb as a mosaic of six different RE types. The data obtained from this study allowed for a comprehensive RE profiling, including the establishment of a library of RE arrays, of the reference mouse genome. Some of these RE arrays may participate in a spectrum of normal and disease biology that are specific for mice.

## Introduction

Over 1,000 whole genomes from various species, ranging from prokaryotes to humans, have been sequenced [1], [2], [3], [4], [5]. The annotated information in these genome databases, which is primarily focused on the protein coding regions in the euchromatin area, are critical assets for current as well as future investigations into biological phenomena [6], [7], [8]. In mice and humans, the entire protein coding sequences are known to constitute ∼2% of the genome and the vast majority of the rest of the genome is presumed to be occupied by various types of repetitive elements (REs) [2], [3], [9], [10].

Current biomedical studies primarily focus on the characterization of the function of genes, both wild-type and polymorphic variant types [11], [12]. A range of molecular and cellular mechanisms, which are presumed to dictate normal as well as disease phenotypes, are almost exclusively explained by the interaction of gene products [13], [14]. The genomes of rodents, non-human primates, and humans are reported to share greater than 85% homology in their protein coding sequences [15], [16], [17], [18], [19]. However, the notion that species-specific phenotypes are predominantly determined by the expression/function of the protein coding sequences may not be consistent with the observation of gross morphological/physical differences between mice and humans, such as a 20 g mouse versus a 70,000 g human.

Recent studies reported that REs participate in a range of normal as well as disease biology, such as differential limb and skull morphology among dog breeds and fragile X syndrome in humans [20], [21], [22]. Some of the tandem REs, in particular tri-nucleotide repeats, may form a secondary structure, such as a stem-loop, which may affect local chromatin configuration and ultimately the genome's higher-order structure [9], [23]. We recently reported the existence of polymorphic RE and RE array (arrangement structure) populations among different human genome databases and postulated that RE arrays may play a role in various biologic processes governing individual-specific phenotype [24].

In this study, to establish a whole-genome profile of REs regarding length, occurrence, and arrangement, the C57BL/6J mouse reference genome was subjected to RE mining analysis using unbiased self-alignment and dot-matrix protocols. Subsequently, the RE array profiles in the sex chromosomes were compared between the reference mouse and human genomes, and one of the most interesting and complex RE arrays in the mouse genome was subjected to deconstruction analysis.

## Results and Discussion

### Unbiased whole-genome profiling of REs: length, occurrence, and arrangement structure

**Figure 1:**
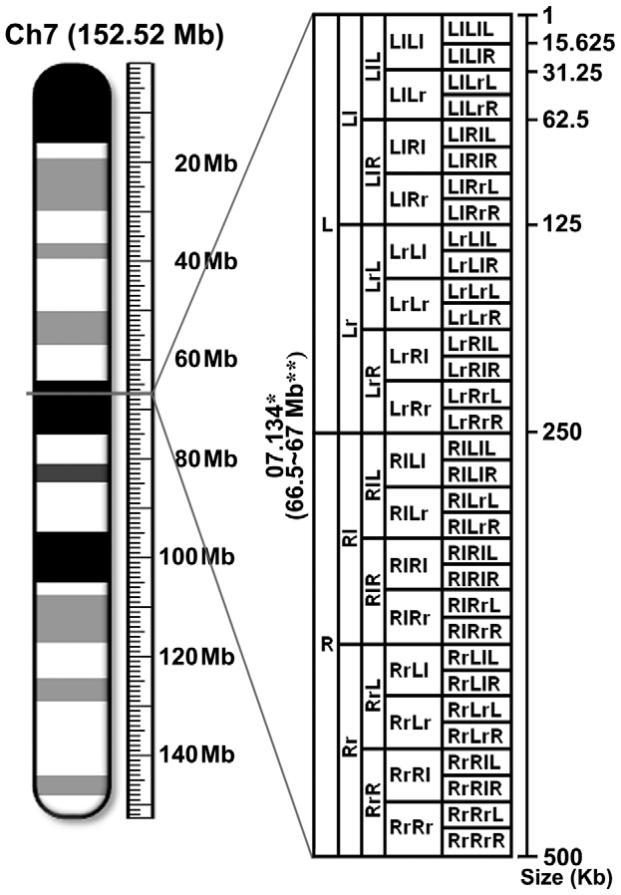
Schedule for fragmentation of a genome unit into sequential half-size subunits. The diagram illustrates how a series of sequential half-size subunits are generated from a genome unit of 0.5 Mb, using the Ch7.134 as an example. During each fragmentation event, the 5′-half is designated as “L" and the 3′-half is designated as “R", and lowercase and uppercase letters are alternated for sequential fragmentation events. The chromosomal ideogram is adopted from the NCBI mouse genome database. *identification of genome unit, **chromosomal location, Ch (chromosome).

The entire mouse (C57BL/6J strain) genome from the National Center for Biotechnology Information (NCBI) was fragmented into 5,321 genome units that were surveyed for REs in regard to length, occurrence, and arrangement structure using an unbiased self-alignment protocol followed by a dot-matrix presentation of the relationships among the RE population (Fig. 1). The unbiased self-alignment protocol in conjunction with the dot-matrix plot display of the REs' relationships allows for an efficient identification of REs (both characterized and uncharacterized) as well as the various forms of arrays formed by the REs [24]. An alternative protocol, which surveys the genome with a biased alignment using previously characterized REs as a probe, may provide a somewhat limited dataset lacking information from the uncharacterized REs and their RE arrays.

**Figure 2:**
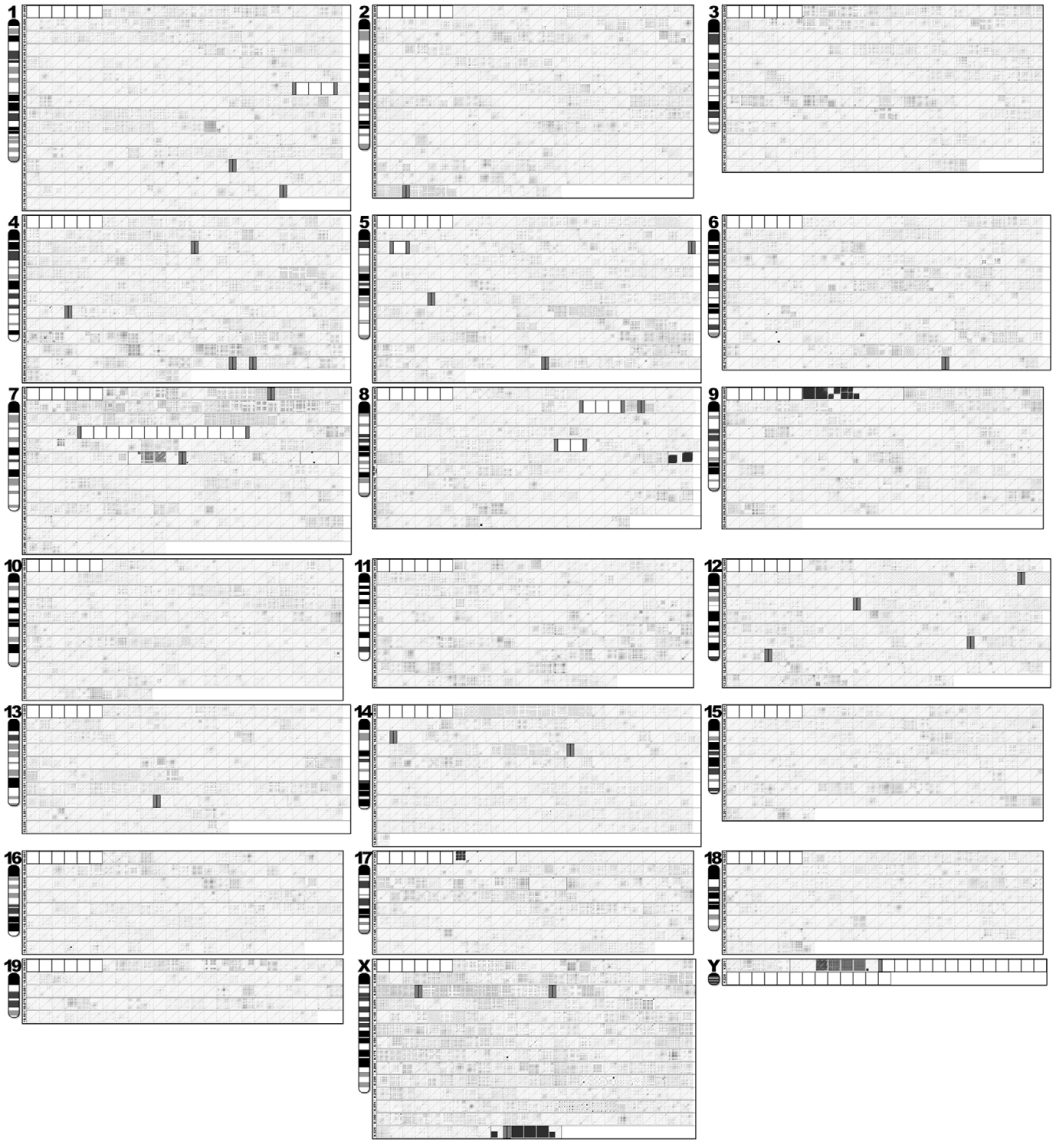
Whole-genome view of occurrence and arrangement structure of REs. The dot-matrix plots of the self-alignment data derived from a total of 5,321 genome units of 0.5 Mb, in addition to 41 subunits of varying sizes, which cover the entire mouse genome, are compiled by chromosome order (1∼19, X, and Y). Each genome unit/subunit is represented by a square and unit identifications are indicated only for the ones on the far left of each row. Genome units without any sequence information (gap) are indicated with a white square. Grey rectangles indicate partial gaps. Ideograms for individual chromosomes, which are adopted from the NCBI mouse genome database, are included as a reference. A set of subunits derived from one genome unit are grouped with a rectangle. A detailed plot view of the RE occurrence and arrangement structure is available through the electronic supplementary figures.

**Figure 3:**
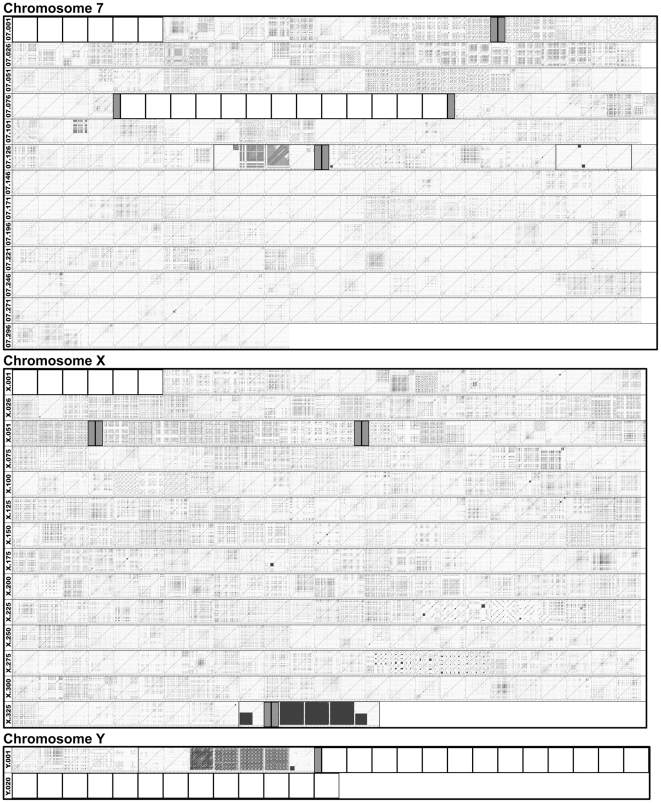
RE arrays of chromosomes 7, X, and Y. The RE arrays plotted from the genome units/subunits of chromosomes 7, X, and Y are assembled in order of 5′ to 3′. The same data sets are presented in a smaller scale in Fig. 2. The same labeling and identification schedules as in Fig. 2 are used. A set of subunits derived from one genome unit are grouped with a rectangle.

The NCBI mouse genome sequence (Build 37.1) was found to be incomplete with substantial gaps lacking sequence information throughout the genome. In particular, 5′-end gaps of ∼3 Mb were present in all chromosomes except for chromosome Y, which only had ∼2.8 Mb sequence information on the 5′-end out of a presumed full-length of ∼16 Mb (Figs. 2 and 3). A large internal gap of 7 Mb was observed on chromosome 7, and chromosomes 1 and 8 had internal gap(s) larger than 1 Mb. Since the RE alignment and corresponding dot-matrix plot data were not retrieved from eight genome units of 0.5 Mb, probably due to the high RE density and/or complexity of the RE arrangement, they were sequentially fragmented into smaller subunits until the data was obtained (Fig. 1).

**Figure 4:**
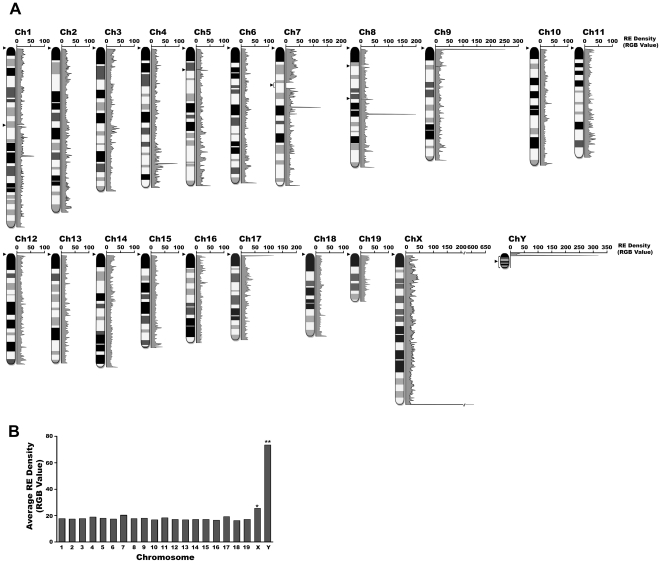
Distribution of average RE density of genome units/subunits of individual chromosomes. **A**. RE density (inverse RGB value) of individual chromosomes. The average RE density of individual genome units/subunits, which are collected as an inverse RGB value, are plotted (plus strand: 5′ to 3′ direction) for each chromosome. Gap regions within each chromosome are indicated with an arrow (≤3 Mb) or arrow with bracket (>3 Mb). **B**. Average RE density of each chromosome. There are significant differences in the profile of RE density among the 21 chromosomes. *chromosome with the highest overall RE density, **chromosome with the highest overall RE density within the limited sequence information available.

A whole-genome survey of the RE mining data revealed that individual chromosomes had unique profiles of REs, primarily in regard to length, density, and arrangement structure (Figs. 2 and 3, and Figs. S1, S2, S3, S4, S5, S6, S7, S8, S9, S10, S11, S12, S13, S14, S15, S16, S17, S18, S19, S20, S21). In particular, unique RE arrays, such as Ch7.032, ChX.241 through ChX.246, and ChY.06LrRlL through ChY.06LrRrR, were observed only in specific chromosomes. It appears that there are more RE arrays with complex configuration in some chromosomes (e.g., chromosomes 7 and X) than the others (e.g., chromosomes 10 and 15). Chromosome-wide analyses of RE occurrence/density, which is presented as the distribution of average dot plot intensity of individual genome units/subunits, revealed that there are substantial differences in RE density within each chromosome as well as among the 21 chromosomes (Fig. 4A). Based on the total average RE density, chromosome Y was determined to be the most densely populated with REs relative to the limited sequence information in the genome database (∼2.8 Mb from the 5′-end of chromosome Y), while chromosome X had the highest overall RE density (Fig. 4B). Interestingly, a series of highly complex RE arrays accompanied by high RE density preceded the major gap of presumed to be ∼13.1 Mb in chromosome Y. It is probable that the high complexity of the RE arrays in this region contributed to the gap, at least in part.

In this study, a whole-genome set of RE profile data regarding length, occurrence, and arrangement structure was compiled for the mouse reference genome using an unbiased self-alignment protocol with the NCBI bl2seq program. However, it needs to be noted that the data presented in this study was generated following fragmentation of the genome into genome units of 0.5 Mb or even smaller subunits, as needed, due to the limitations of the tool employed (web-based NCBI bl2seq program). The development of an on-site tool capable of processing an entire chromosome and/or whole genome as a single query may be needed to obtain a more comprehensive data set accounting for all potential RE occurrences and relationships within a chromosome/genome compared to one from the sequential analysis of fragmented genome units.

### Structural characteristics of selective RE arrays based on dot-matrix plots

**Figure 5:**
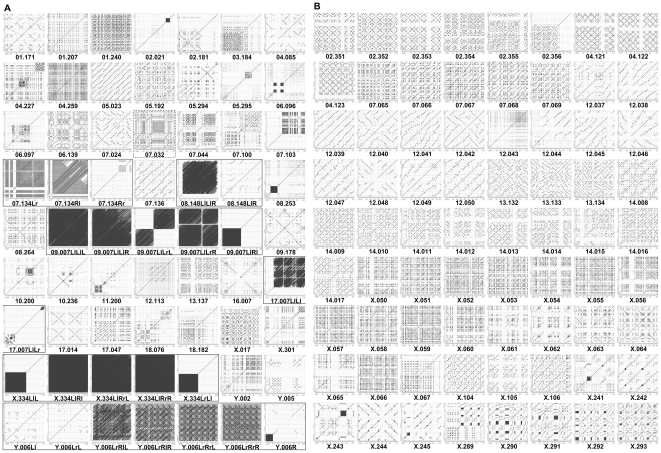
Compilation of unique RE arrays selected throughout the entire genome. A total of 135 unique RE arrays are selected throughout the mouse genome and compiled by chromosome order (1∼19, X, and Y). **A**. Compilation of RE arrays from a single genome unit or a set of subunits from one genome unit. A set of subunits derived from one genome unit is indicated by a rectangle. The Ch7.032 genome unit is highlighted by a dotted square. **B**. Compilation of RE arrays spanning consecutive genome units.

A total of 135 RE arrays, which retain complex and/or unique architectural configurations, were selected following a survey of the whole-genome set of dot-matrix plot data (Figs. 2, 3 and 5). The RE arrays, which spanned in consecutive genome units of more than one, were compiled separately to demonstrate their length/extent (Fig. 5B). These individual RE arrays were constructed by combinations of multiple RE types with varying lengths, occurrences, and orientations (direct vs. inverse). It is possible that some of the larger REs, which were represented by longer line plots, harbor mosaic arrangements of a specific set of smaller REs derived from different RE types. Interestingly, some of the RE arrays, such as Ch7.032, Ch7.134Rl, Ch17.007LlLl, ChX.017, and ChY.006LrRrR, were complex, but they also seemed to be uniquely organized (Fig. 5A).

Simple RE arrays can be configured by the interpretation of the physical characteristics of the dots and lines on the two-dimensional dot-matrix plot. For instance, an RE plot formed by the crossing of two lines in opposite orientations (e.g., Ch17.014 in Fig. 5A) indicates a palindromic structure, while a square or rectangle box filled with lines of the same orientation (e.g., Ch9.007LlRl in Fig. 5A) represents a tandem repeat region. However, the apparent high level complexity of certain RE arrays (e.g., Ch7.032, ChX.017, ChY.006LrRrR in Fig. 5A) did not allow for a linear deconstruction of the arrangement details by a brief examination of the physical properties (e.g., length, occurrence, orientation) of dots and lines. For an efficient deconstruction of these highly complex RE arrays, a software tool, which is capable of translating the data embedded in the individual dots and lines onto a scaled line map, needs to be developed.

Interestingly, specific regions in certain genome units/subunits were almost free of RE dots and lines and some of them were surrounded by densely populated RE arrays (Figs. 3 and 5A). One possibility is that the RE-free regions are populated with protein coding sequences. The data obtained from this study demonstrated that REs are often arranged in a complex, but well-organized, manner, generating a diverse population of RE arrays, which may contribute to the higher-order architecture specific for the mouse genome.

### Comparative analysis of the RE array profiles in the chromosomes X and Y: mouse vs. human

**Figure 6:**
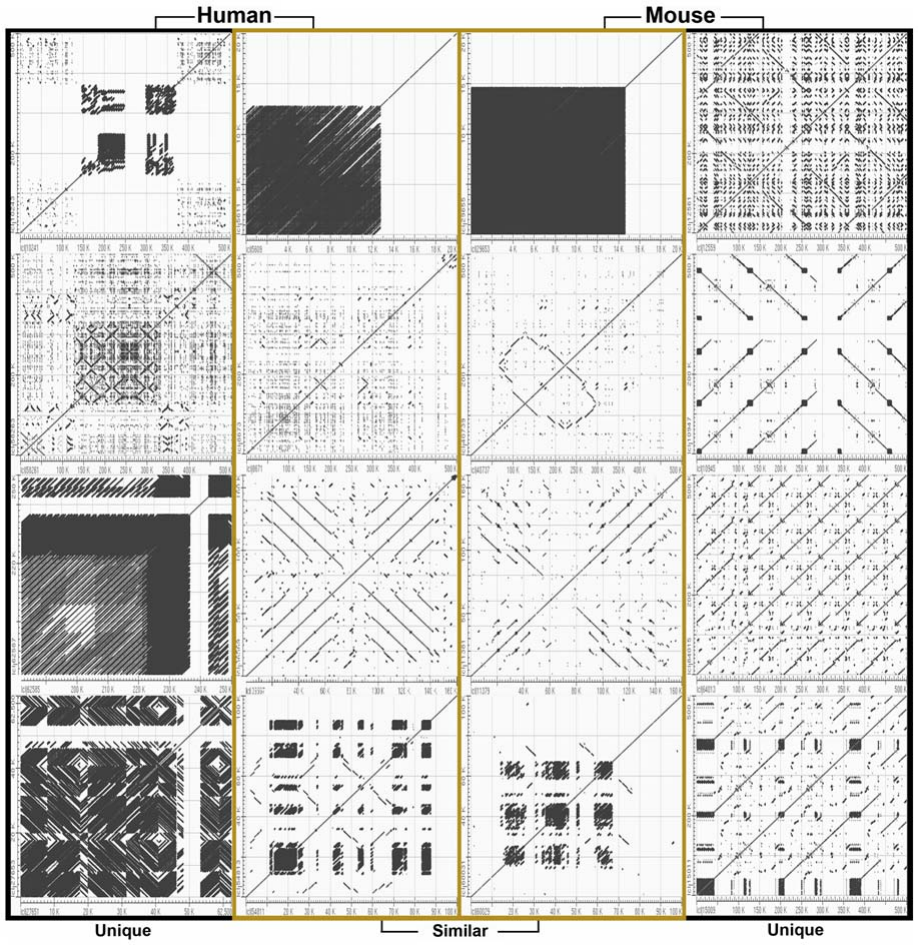
Unique and similar RE arrays in chromosomes X and Y: mouse vs. human. The RE arrays identified in the chromosomes X and Y of the reference mouse genome were compared to those from the reference human genome. Four RE arrays unique for the mouse and human chromosomes were selected. Also, four similar RE arrays were identified from the mouse and human chromosomes.

The human and mouse genomes are reported to share close to 99% of their gene population and ∼85% of each genome can be partitioned into corresponding regions in a synteny map based on the gene sequences shared between these two species [3]. To determine whether the RE array profile of the mouse genome is different from the human genome, the RE arrays identified in the chromosomes X and Y of the reference mouse genome were compared to those from the reference human genome. Overall, each species had its unique profile of RE arrays in the chromosomes X and Y; however, it appeared that some mouse RE arrays share basic structural configurations with certain human RE arrays (Fig. 6) [24]. The data obtained from this study indicates that in contrast to the high similarity in gene sequences between the mouse and human, the RE array population of the mouse genome is only distantly related to one of the human genome. It is possible that the species-specific profiles of REs and RE arrays are associated with the fine-tuning of gene expression that contributes to the differential phenotypes observed between mouse and human.

### Translocation between the tandem RE array of the immunoglobulin M (IgM) switch region and c-*Myc* gene

**Figure 7:**
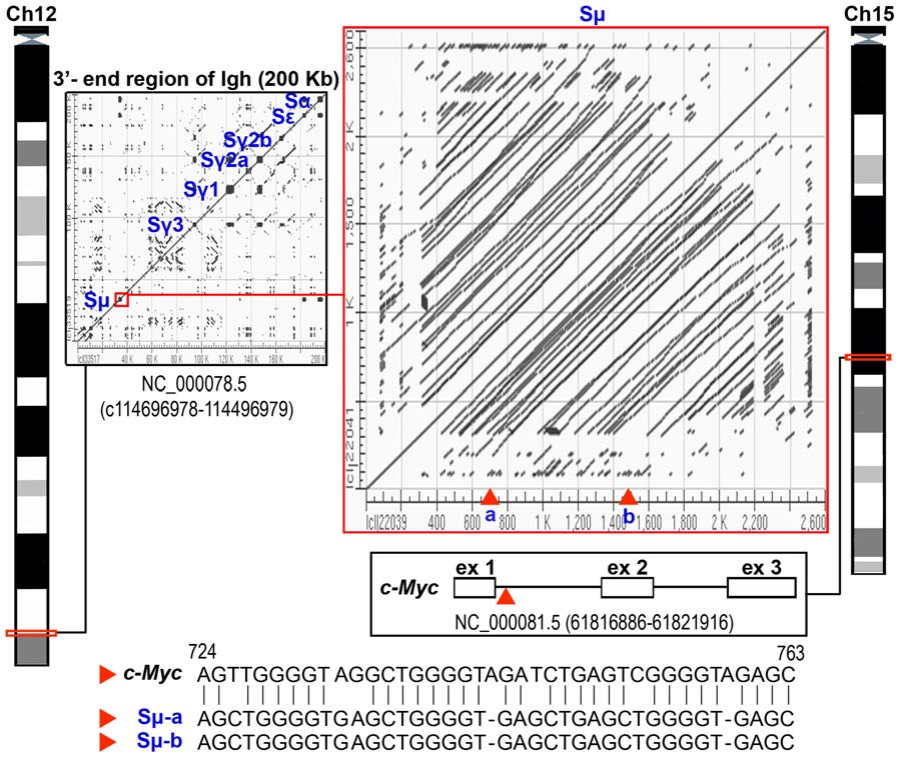
Translocation between the tandem RE array of the IgM switch region and the c-*Myc* gene. The 3′-end of the immunoglobulin heavy chain (IGh) is presented as a dot-matrix plot with markings for the various switch regions (in blue). The zoomed-in tandem RE array dot-matrix plot represents the IgM switch region (Sμ). The illustration, involving Sμ and c-*Myc* gene sequences, depicts the putative translocation event between two sections of the IgM switch region on chromosome 12 and a 40-nucleotide stretch within the c-*Myc* gene on chromosome 15. One unique stretch of 40 nucleotides in the c-*Myc* sequence, corresponding to a reported translocation breakpoint in the c-*Myc* gene, had a high homology (80% identity: 5 mismatches and 2 gaps) with two sections of the IgM switch region tandem array. Ch (mouse chromosome), ex (exon), red triangles (putative translocation breakpoints).

A literature survey was performed to identify the potential for an RE array's involvement in the translocation between different mouse chromosomes. One well-described translocation event occurred between the IgM switch region on chromosome 12 and the c-*Myc* gene on chromosome 15 [25]. The IgM switch region is a tandem RE array which is formed with ∼150 repeats of a short stretch of homologous sequences [26]. The c-*Myc* gene sequence was surveyed with the IgM switch region sequence to identify a locus which may serve as a breakpoint for the translocation between the IgM tandem RE array region and the c-*Myc* gene (Fig. 7). Interestingly, one unique stretch of 40 nucleotides in the c-*Myc* sequence had a homologous alignment (80% identity; 5 mismatches and 2 gaps) with two sections of the IgM switch region. In fact, the 40 nucleotide-stretch was localized to a reported translocation breakpoint in the c-*Myc* gene (GenBank No.: K01873.1) [27]. The findings from this study suggest that the sequence and arrangement characteristics of the IgM switch region RE array may play a crucial role in the specific translocation into the c-*Myc* locus.

### Linear deconstruction of RE arrangement structures in the Ch7.032 genome unit

**Figure 8:**
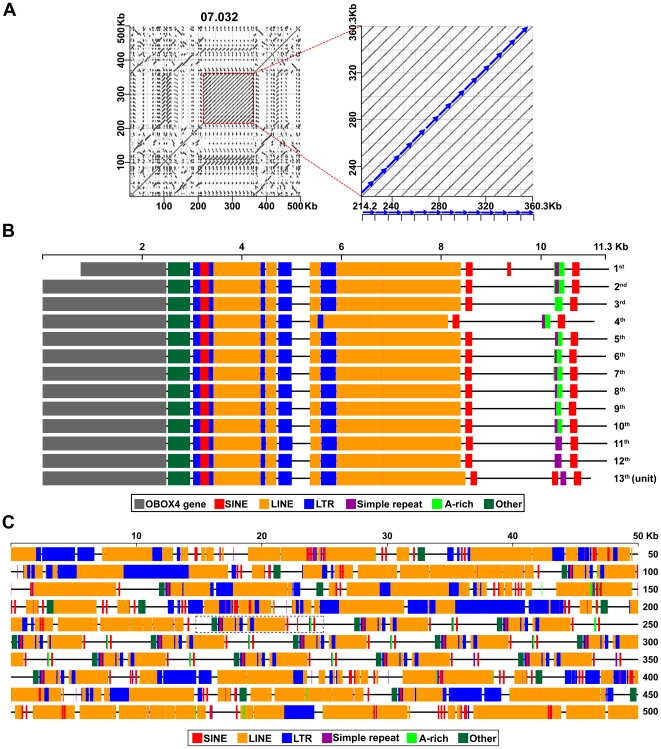
Line mapping of REs/genes within the Ch7.032 genome unit. **A**. The dot-matrix plot is derived from an unbiased self-alignment of the Ch7.032 genome unit. The REs in the area highlighted with a dotted red line are subjected to a line mapping experiment. **B**. The RE profile within the tandem 13 repeats of the ∼11.3 Kb unit in the highlighted structure are plotted on a scaled line map using color coding to identify RE types. In addition, *Obox4* gene sequences are marked within each repeat unit. **C**. The RE profile of the entire Ch7.032 genome unit of 0.5 Mb are plotted on a scaled line map for all RE types with color coding identification. The dotted rectangle represents the location of the first ∼11.3 Kb repeat unit within the Ch7.032 genome unit. High-resolution line maps (for maps on panels B and C) are accessible through Figs. S22 and S23.

Among a number of the complex, ordered, and unique RE arrays, the dot-matrix plot of the Ch7.032 genome unit, which resembles a circuit board full of microprocessors, was selected for an experiment to deconstruct the RE arrays. Initially, among the RE arrays found in the Ch7.032 genome unit, the lined square was isolated and subjected to RE profiling analysis (Fig. 8A). Examination of the corresponding alignment data for the lines in the square revealed that it is a tandem repeat structure, harboring 13 repeats of an ∼11.3 Kb unit. A survey of the individual ∼11.3 Kb repeat units for RE profiles using RepeatMasker [28] and its RE probe library identified several different RE types: long terminal repeat (LTR), long interspersed nuclear element (LINE), short interspersed nuclear element (SINE), A-rich region, GA tandem repeats, and others. In addition, an open reading frame analysis to search for putative protein coding sequences in the repeat unit revealed the presence of a full-coding region for the *Obox4* (oocyte-specific homeobox 4) gene [29], [30], [31]. Subsequently, the RE profile data of the entire Ch7.032 genome unit (0.5 Mb) was obtained using the same RepeatMasker protocol that was used for the tandem repeat structure.

For line mapping of the RE and/or gene profiles, relevant information, such as coordinates and orientations of RE alignments as well as genes, was extracted. Subsequently, a scaled line map was plotted for individual RE and/or gene occurrences. The line maps for the individual REs and/or genes were then merged to compile the entire data set of RE/gene profiles onto one line map using a layer function. Consequently, the occurrence of each RE/gene type can be viewed individually on a line map by separation of the specific layer. Using this protocol, a scaled line map of REs and genes was generated for each ∼11.3 Kb repeat unit (Fig. 8B and Fig. S22). Eleven of the 13 repeat units retained a full-coding potential of 453 amino acids for the *Obox4* gene. The 5′-end of the first repeat unit was mapped to the middle of the *Obox4* gene and the seventh repeat unit retained a partial coding potential of 439 amino acids due to premature stop linked to a deletion of five nucleotides near the 3′-end of the *Obox4* gene. According to the latest reference sequence of the *Obox4* gene, it is 1.939 Kb long and consists of three exons and two introns; however, the 3′-end of the exon 3 has not been clearly defined [29]. Based on the identification of a putative poly (A) signal downstream of the stop codon, we now propose that the *Obox4* gene is 2.484 Kb. Subsequently, the RE line map of the entire Ch7.032 genome unit (0.5 Mb) was constructed using the same RepeatMasker protocol that was used for the generation of the tandem structure line map (Fig. 8C and Fig. S23). In this study, it was demonstrated that an interactive line map of the RE and/or gene profiles can be efficiently assembled for RE arrays, which were identified by self-alignment analyses, using readily available computer programs and the RepeatMasker database. However, the development of a new tool may be necessary for a rapid deconstruction of complex RE arrays in conjunction with the establishment of a more comprehensive set of RE probe libraries.

In conclusion, a whole-genome library of the mouse RE arrays, some of which were complex and well-organized, was established using the data obtained from unbiased self-alignments of 5,321 genome units followed by dot-matrix plotting. Each chromosome had a unique RE profile in regard to density and arrangement structure. In addition, the RE array profile of the mouse genome was unique in comparison to the one from the human genome. It can be speculated that some of these REs and RE arrays play a role in the genome's higher-order structure, and consequently in cell division, gene expression, and/or other biologic activities, including species-specific phenotype determination.

## Materials and Methods

### Generation of a library of mouse genome units of 0.5 Mb

A library of 5,321 genome units was generated for the entire mouse (C57BL/6J strain) genome from National Center for Biotechnology Information (NCBI) (Build 37.1) by sequential *in silico* cutting at every 0.5 Mb, starting at the 5′-end of each chromosome (1 through 19, X, and Y) [3], [32]. The genome unit of 0.5 Mb conforms to the query size limit of the NCBI bl2seq program [33], which was employed in this study for an unbiased self-alignment analyses. The genome units were processed and stored using the Lasergene (version 8.0.2) program (DNASTAR, Madison, WI), a DNA editing and alignment program, and the gaps (no sequence information) in the NCBI genome database were filled with “N"s to maintain the size of each genome unit. Each genome unit of 0.5 Mb was labeled with an identifier (e.g., the 25^th^ genome unit of chromosome 7: Ch7.025).

### Sequential fragmentation of a genome unit of 0.5 Mb

For the genome units of 0.5 Mb which could not be processed by the NCBI bl2seq program due to the high RE density and/or RE arrangement complexity, each of these genome units was sequentially cut in half to generate a range of smaller genome subunits until the self-alignment data could be processed by the bl2seq program. Each genome subunit was identified by the schedule described in Fig. 1 (e.g., the first half and second half of Ch7.025 were designated as Ch7.025L and Ch7.025R, respectively).

### Survey of REs in regard to length, occurrence, and arrangement structure within each genome unit/subunit

Individual genome units/subunits were subjected to an unbiased self-alignment analyses (alignment of two identical sequences) using the bl2seq program, which was originally designed to identify homology between two different sequences [33]. Two different result formats are expected to be yielded from the bl2seq alignment analysis: pair-wise individual RE sequence alignments and corresponding dot-matrix plots [24]. The dot-matrix plot images of individual genome units/subunits, which represent the relationships among the RE population in each genome unit/subunit, were saved as a png file within the Photoshop program (Adobe Systems Inc., San Jose, CA).

### Measurement of RE density in dot-matrix plot images of individual genome units/subunits

The average RE density was measured from the dot-matrix plots of individual genome units/subunits as an average RGB value using the histogram function of the Photoshop program (Adobe Systems Inc.) [24]. Since the RGB values obtained are inversely correlated with the density of RE dot-matrix plots (the higher RGB value, the lower RE density), they were subtracted from the maximum RGB value of 255 (white) prior to graphic presentation of the average RE density of individual genome units/subunits. To compensate the RGB values obtained from the genome unit (0.5 Mb) versus the smaller subunits, the following protocols were implemented: 1) the smallest genome subunit was identified as 15.625 Kb and 2) the average RE densities of the genome units/subunits greater than 15.625 Kb were normalized to the 15.625 Kb subunit following the identification of specific conversion factors. The specific conversion factor was calculated for each genome unit/subunit from a standard curve (a set of genome unit and subunits [15.625 Kb ∼500 Kb] vs. respective RE densities), which was generated using a full set of genome unit-subunits from four-0.5 Mb genome units with relatively even RE distribution.

### Line mapping of different RE types in the Ch7.032 RE array

The Ch7.032 genome unit of 0.5 Mb, which displayed one of the most complex and unique dot-matrix plot patterns, was surveyed to identify REs using a library of RE probes within RepeatMasker [28]. In addition, the ∼11.3 Kb units of the tandem 13-repeat array within the Ch7.032 genome unit were subjected to an open reading frame analyses to search for putative protein coding sequences using the Lasergene (version 8.0.2) program (DNASTAR). Following color designation for individual RE types and/or genes, their occurrences were line-drawn on a scale and the individual line-drawn maps representing different RE types and/or genes were merged to compile the entire data set using the layer function of the Photoshop program (Adobe Systems Inc.).

### Comparison of RE arrays in the chromosomes X and Y: mouse vs. human

Using the same protocol, which was applied for the identification of RE arrays in the mouse chromosomes X and Y, the human chromosomes X and Y (NCBI Build 37.1) were surveyed for RE arrays. Subsequently, the RE array population from the mouse chromosomes was visually compared to the one from the human chromosomes.

## Supporting Information

Figure S1 **Detailed dot-matrix plot view of the RE arrays in the mouse chromosome 1 from** **Fig. 2** **.** The dot-matrix plots of the self-alignment data derived from a total of 395 genome units of 0.5 Mb are compiled for the mouse chromosome 1. Each genome unit is represented by a square and unit identifications are indicated only for the ones on the far left of each row. Genome units without any sequence information (gap) are indicated with a white square. Grey rectangles indicate partial gaps.
(TIF)

Figure S2 **Detailed dot-matrix plot view of the RE arrays in the mouse chromosome 2 from** **Fig. 2** **.** The dot-matrix plots of the self-alignment data derived from a total of 364 genome units of 0.5 Mb are compiled for the mouse chromosome 2. Each genome unit is represented by a square and unit identifications are indicated only for the ones on the far left of each row. Genome units without any sequence information (gap) are indicated with a white square. Grey rectangles indicate partial gaps.
(TIF)

Figure S3 **Detailed dot-matrix plot view of the RE arrays in the mouse chromosome 3 from** **Fig. 2** **.** The dot-matrix plots of the self-alignment data derived from a total of 320 genome units of 0.5 Mb are compiled for the mouse chromosome 3. Each genome unit is represented by a square and unit identifications are indicated only for the ones on the far left of each row. Genome units without any sequence information (gap) are indicated with a white square.
(TIF)

Figure S4 **Detailed dot-matrix plot view of the RE arrays in the mouse chromosome 4 from** **Fig. 2** **.** The dot-matrix plots of the self-alignment data derived from a total of 312 genome units of 0.5 Mb are compiled for the mouse chromosome 4. Each genome unit is represented by a square and unit identifications are indicated only for the ones on the far left of each row. Genome units without any sequence information (gap) are indicated with a white square. Grey rectangles indicate partial gaps.
(TIF)

Figure S5 **Detailed dot-matrix plot view of the RE arrays in the mouse chromosome 5 from** **Fig. 2** **.** The dot-matrix plots of the self-alignment data derived from a total of 306 genome units of 0.5 Mb are compiled for the mouse chromosome 5. Each genome unit is represented by a square and unit identifications are indicated only for the ones on the far left of each row. Genome units without any sequence information (gap) are indicated with a white square. Grey rectangles indicate partial gaps.
(TIF)

Figure S6 **Detailed dot-matrix plot view of the RE arrays in the mouse chromosome 6 from** **Fig. 2** **.** The dot-matrix plots of the self-alignment data derived from a total of 300 genome units of 0.5 Mb are compiled for the mouse chromosome 6. Each genome unit is represented by a square and unit identifications are indicated only for the ones on the far left of each row. Genome units without any sequence information (gap) are indicated with a white square. Grey rectangles indicate partial gaps.
(TIF)

Figure S7 **Detailed dot-matrix plot view of the RE arrays in the mouse chromosome 7 from** **Fig. 2** **.** The dot-matrix plots of the self-alignment data derived from a total of 306 genome units of 0.5 Mb are compiled for the mouse chromosome 7. Each genome unit/subunit is represented by a square and unit identifications are indicated only for the ones on the far left of each row. Genome units without any sequence information (gap) are indicated with a white square. Grey rectangles indicate partial gaps. A set of subunits derived from one genome unit are grouped with a rectangle.
(TIF)

Figure S8 **Detailed dot-matrix plot view of the RE arrays in the mouse chromosome 8 from** **Fig. 2** **.** The dot-matrix plots of the self-alignment data derived from a total of 264 genome units of 0.5 Mb are compiled for the mouse chromosome 8. Each genome unit/subunit is represented by a square and unit identifications are indicated only for the ones on the far left of each row. Genome units without any sequence information (gap) are indicated with a white square. Grey rectangles indicate partial gaps. A set of subunits derived from one genome unit are grouped with a rectangle.
(TIF)

Figure S9 **Detailed dot-matrix plot view of the RE arrays in the mouse chromosome 9 from** **Fig. 2** **.** The dot-matrix plots of the self-alignment data derived from a total of 249 genome units of 0.5 Mb are compiled for the mouse chromosome 9. Each genome unit/subunit is represented by a square and unit identifications are indicated only for the ones on the far left of each row. Genome units without any sequence information (gap) are indicated with a white square. A set of subunits derived from one genome unit are grouped with a rectangle.
(TIF)

Figure S10 **Detailed dot-matrix plot view of the RE arrays in the mouse chromosome 10 from** **Fig. 2** **.** The dot-matrix plots of the self-alignment data derived from a total of 260 genome units of 0.5 Mb are compiled for the mouse chromosome 10. Each genome unit is represented by a square and unit identifications are indicated only for the ones on the far left of each row. Genome units without any sequence information (gap) are indicated with a white square.
(TIF)

Figure S11 **Detailed dot-matrix plot view of the RE arrays in the mouse chromosome 11 from** **Fig. 2** **.** The dot-matrix plots of the self-alignment data derived from a total of 244 genome units of 0.5 Mb are compiled for the mouse chromosome 11. Each genome unit is represented by a square and unit identifications are indicated only for the ones on the far left of each row. Genome units without any sequence information (gap) are indicated with a white square.
(TIF)

Figure S12 **Detailed dot-matrix plot view of the RE arrays in the mouse chromosome 12 from** **Fig. 2** **.** The dot-matrix plots of the self-alignment data derived from a total of 243 genome units of 0.5 Mb are compiled for the mouse chromosome 12. Each genome unit is represented by a square and unit identifications are indicated only for the ones on the far left of each row. Genome units without any sequence information (gap) are indicated with a white square. Grey rectangles indicate partial gaps.
(TIF)

Figure S13 **Detailed dot-matrix plot view of the RE arrays in the mouse chromosome 13 from** **Fig. 2** **.** The dot-matrix plots of the self-alignment data derived from a total of 241 genome units of 0.5 Mb are compiled for the mouse chromosome 13. Each genome unit is represented by a square and unit identifications are indicated only for the ones on the far left of each row. Genome units without any sequence information (gap) are indicated with a white square. Grey rectangles indicate partial gaps.
(TIF)

Figure S14 **Detailed dot-matrix plot view of the RE arrays in the mouse chromosome 14 from** **Fig. 2** **.** The dot-matrix plots of the self-alignment data derived from a total of 251 genome units of 0.5 Mb are compiled for the mouse chromosome 14. Each genome unit is represented by a square and unit identifications are indicated only for the ones on the far left of each row. Genome units without any sequence information (gap) are indicated with a white square. Grey rectangles indicate partial gaps.
(TIF)

Figure S15 **Detailed dot-matrix plot view of the RE arrays in the mouse chromosome 15 from** **Fig. 2** **.** The dot-matrix plots of the self-alignment data derived from a total of 207 genome units of 0.5 Mb are compiled for the mouse chromosome 15. Each genome unit/subunit is represented by a square and unit identifications are indicated only for the ones on the far left of each row. Genome units without any sequence information (gap) are indicated with a white square.
(TIF)

Figure S16 **Detailed dot-matrix plot view of the RE arrays in the mouse chromosome 16 from** **Fig. 2** **.** The dot-matrix plots of the self-alignment data derived from a total of 197 genome units of 0.5 Mb are compiled for the mouse chromosome 16. Each genome unit is represented by a square and unit identifications are indicated only for the ones on the far left of each row. Genome units without any sequence information (gap) are indicated with a white square.
(TIF)

Figure S17 **Detailed dot-matrix plot view of the RE arrays in the mouse chromosome 17 from** **Fig. 2** **.** The dot-matrix plots of the self-alignment data derived from a total of 191 genome units of 0.5 Mb are compiled for the mouse chromosome 17. Each genome unit/subunit is represented by a square and unit identifications are indicated only for the ones on the far left of each row. Genome units without any sequence information (gap) are indicated with a white square. A set of subunits derived from one genome unit are grouped with a rectangle.
(TIF)

Figure S18 **Detailed dot-matrix plot view of the RE arrays in the mouse chromosome 18 from** **Fig. 2** **.** The dot-matrix plots of the self-alignment data derived from a total of 182 genome units of 0.5 Mb are compiled for the mouse chromosome 18. Each genome unit is represented by a square and unit identifications are indicated only for the ones on the far left of each row. Genome units without any sequence information (gap) are indicated with a white square.
(TIF)

Figure S19 **Detailed dot-matrix plot view of the RE arrays in the mouse chromosome 19 from** **Fig. 2** **.** The dot-matrix plots of the self-alignment data derived from a total of 123 genome units of 0.5 Mb are compiled for the mouse chromosome 19. Each genome unit is represented by a square and unit identifications are indicated only for the ones on the far left of each row. Genome units without any sequence information (gap) are indicated with a white square.
(TIF)

Figure S20 **Detailed dot-matrix plot view of the RE arrays in the mouse chromosome X from** **Fig. 2** **.** The dot-matrix plots of the self-alignment data derived from a total of 334 genome units of 0.5 Mb are compiled for the mouse chromosome X. Each genome unit/subunit is represented by a square and unit identifications are indicated only for the ones on the far left of each row. Genome units without any sequence information (gap) are indicated with a white square. Grey rectangles indicate partial gaps. A set of subunits derived from one genome unit are grouped with a rectangle.
(TIF)

Figure S21 **Detailed dot-matrix plot view of the RE arrays in the mouse chromosome Y from** **Fig. 2** **.** The dot-matrix plots of the self-alignment data derived from a total of 32 genome units of 0.5 Mb are compiled for the mouse chromosome Y. Each genome unit/subunit is represented by a square and unit identifications are indicated only for the ones on the far left of each row. Genome units without any sequence information (gap) are indicated with a white square. A set of subunits derived from one genome unit are grouped with a rectangle.
(TIF)

Figure S22 **High-resolution view of line maps for REs/genes within the Ch7.032 genome unit (** **Fig. 8B** **).** The high resolution image file provides details of the line map presented in Fig. 8B. In the case of an overlap of more than one nucleotide between two REs, the 3′-end of the upstream RE is indicated by a solid line while the 5′-end of the downstream RE is indicated by a dotted line; however, only solid lines are used when the overlap is one nucleotide. To differentiate a no gap from a one nucleotide overlap, a dotted line is preceded by a solid line to indicate the start and end of two REs. Individual RE and/or gene types can be viewed separately on a line map using the layer function within the Photoshop program (Adobe Systems Inc.).
(TIF)

Figure S23 **High-resolution view of line maps for REs/genes within the Ch7.032 genome unit (** **Fig. 8C** **).** The high resolution image file provides details of the line map presented in Fig. 8C. In the case of an overlap of more than one nucleotide between two REs, the 3′-end of the upstream RE is indicated by a solid line while the 5′-end of the downstream RE is indicated by a dotted line; however, only solid lines are used when the overlap is one nucleotide. To differentiate a no gap from a one nucleotide overlap, a dotted line is preceded by a solid line to indicate the start and end of two REs. Individual RE and/or gene types can be viewed separately on a line map using the layer function within the Photoshop program (Adobe Systems Inc.).
(TIF)
