# Supplementary figures and images for: Unique Profile of Ordered Arrangements of Repetitive Elements in the C57BL/6J Mouse Genome Implicating Their Functional Roles
Source: PLoS One. 2012 Apr 18;7(4):e35156. doi: 10.1371/journal.pone.0035156 (PMC3329453; doi:10.1371/journal.pone.0035156)

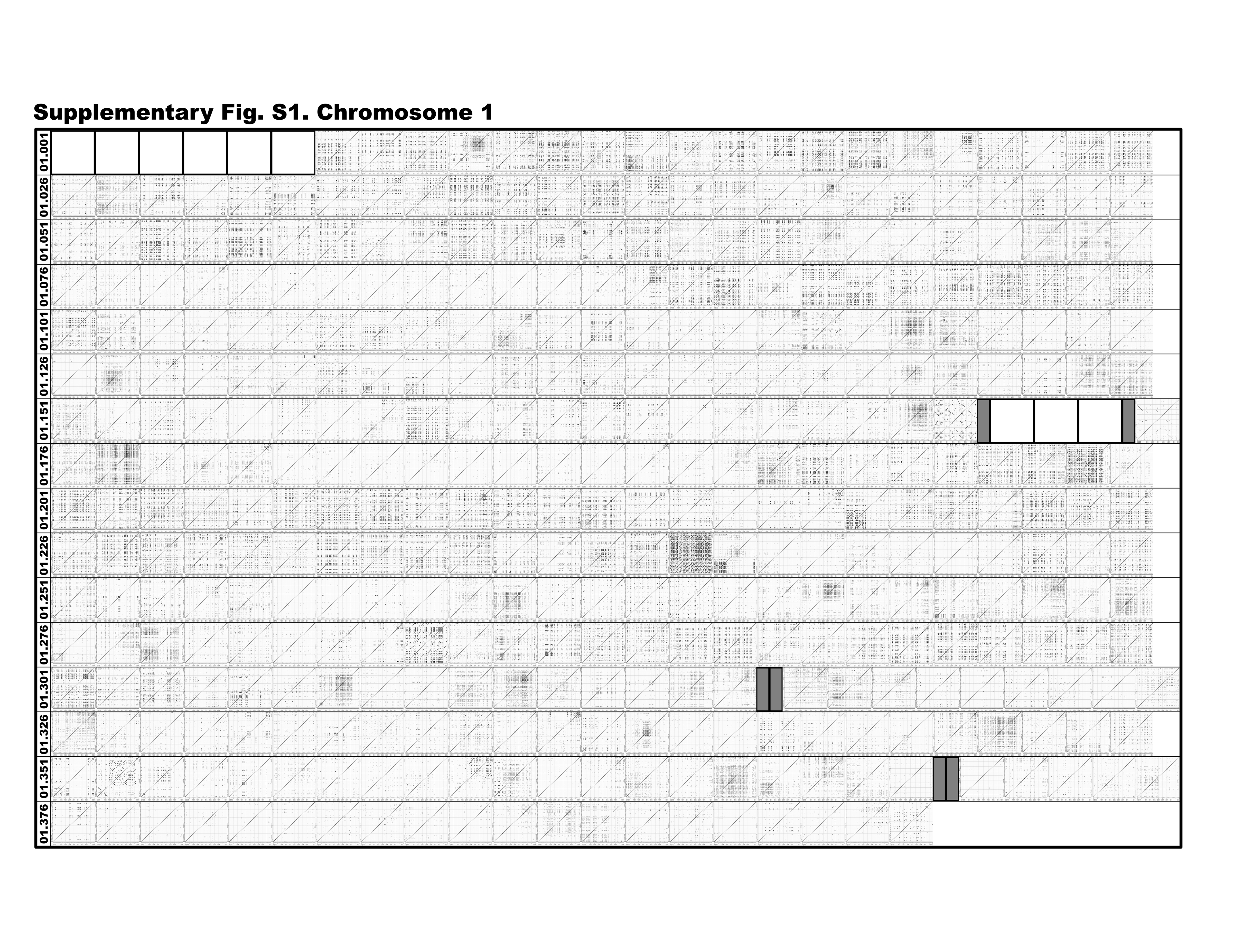

Supplement: Figure S1 — Detailed dot-matrix plot view of the RE arrays in the mouse chromosome 1 from Fig. 2 . The dot-matrix plots of the self-alignment data derived from a total of 395 genome units of 0.5 Mb are compiled for the mouse chromosome 1. Each genome unit is represented by a square and unit identifications are indicated only for the ones on the far left of each row. Genome units without any sequence information (gap) are indicated with a white square. Grey rectangles indicate partial gaps. (TIF) [file pone.0035156.s001.tif]

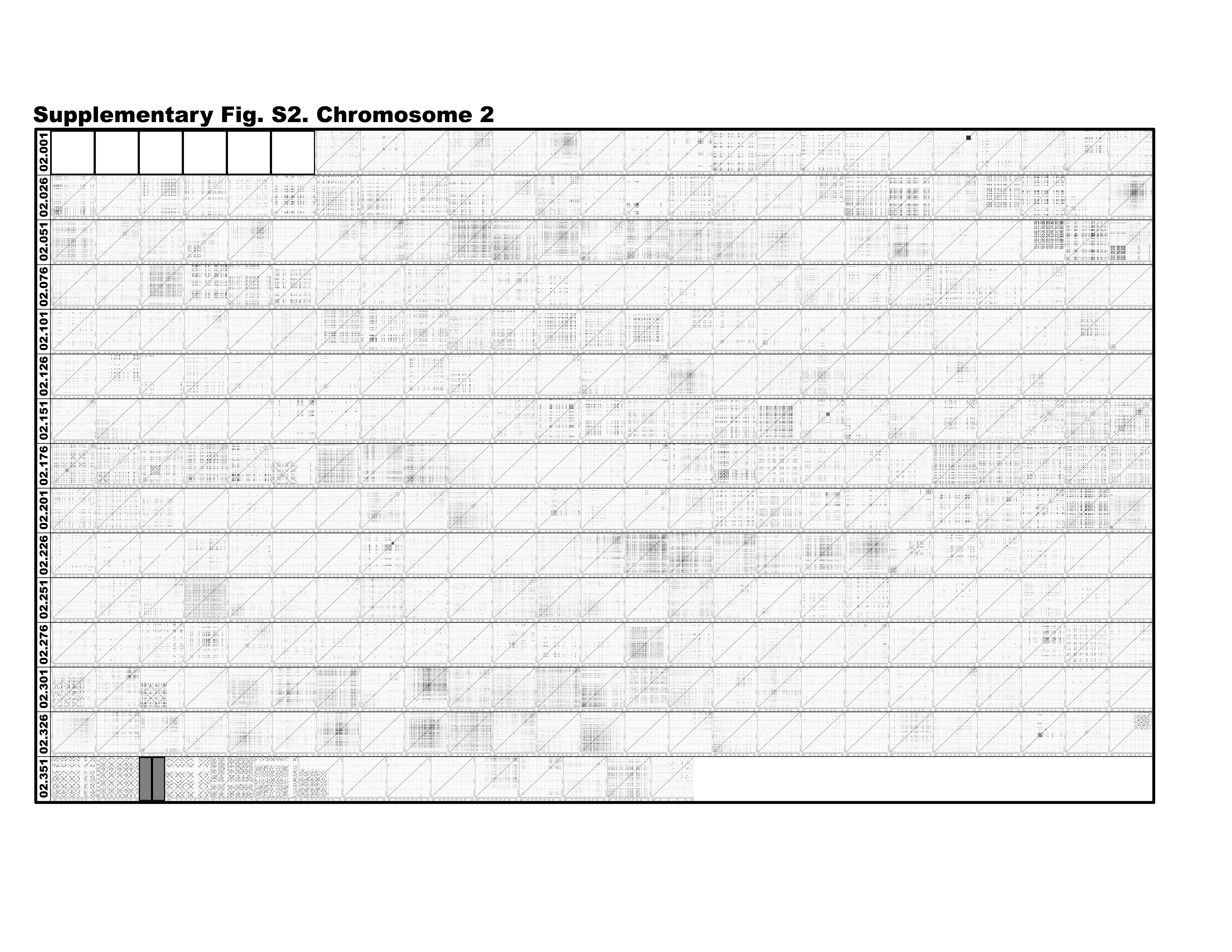

Supplement: Figure S2 — Detailed dot-matrix plot view of the RE arrays in the mouse chromosome 2 from Fig. 2 . The dot-matrix plots of the self-alignment data derived from a total of 364 genome units of 0.5 Mb are compiled for the mouse chromosome 2. Each genome unit is represented by a square and unit identifications are indicated only for the ones on the far left of each row. Genome units without any sequence information (gap) are indicated with a white square. Grey rectangles indicate partial gaps. (TIF) [file pone.0035156.s002.tif]

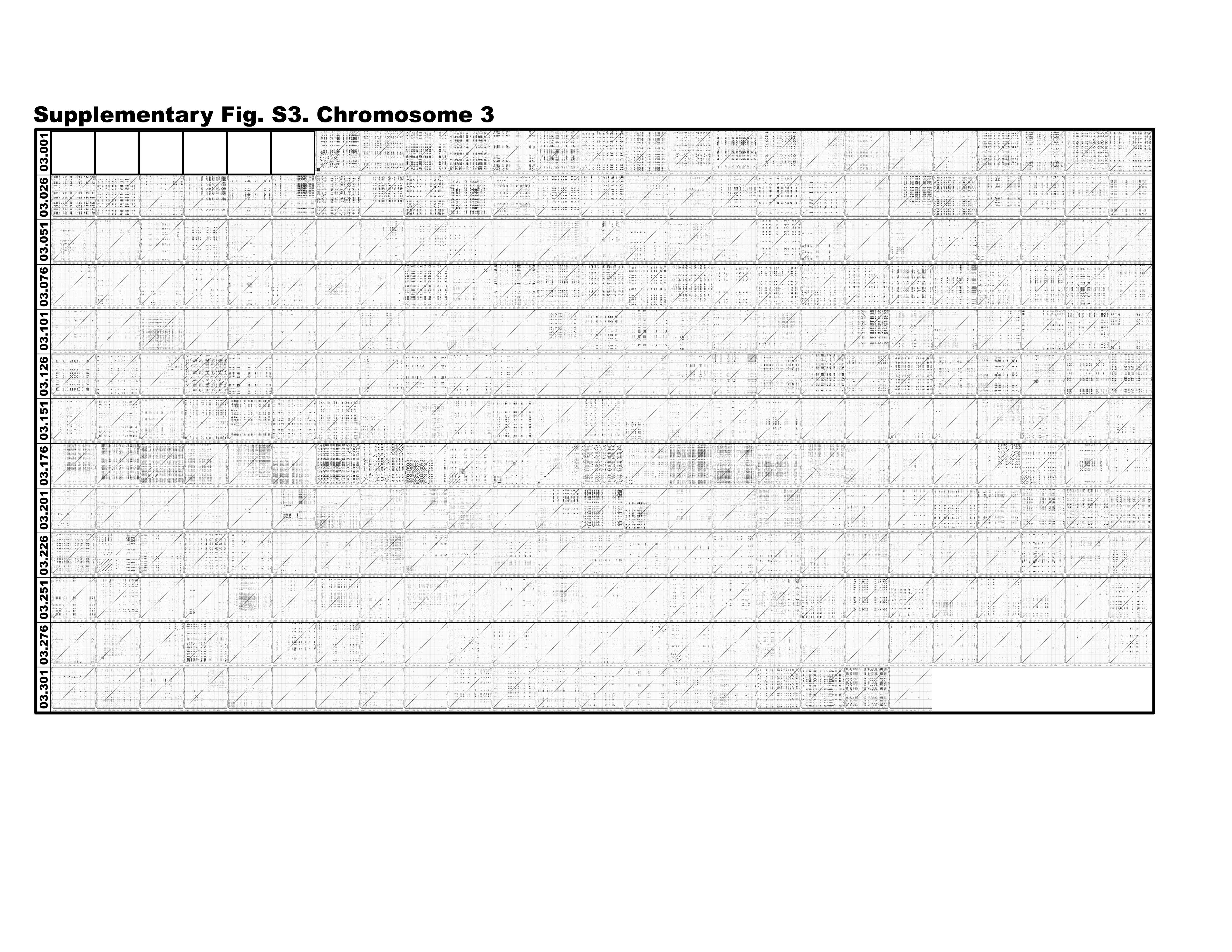

Supplement: Figure S3 — Detailed dot-matrix plot view of the RE arrays in the mouse chromosome 3 from Fig. 2 . The dot-matrix plots of the self-alignment data derived from a total of 320 genome units of 0.5 Mb are compiled for the mouse chromosome 3. Each genome unit is represented by a square and unit identifications are indicated only for the ones on the far left of each row. Genome units without any sequence information (gap) are indicated with a white square. (TIF) [file pone.0035156.s003.tif]

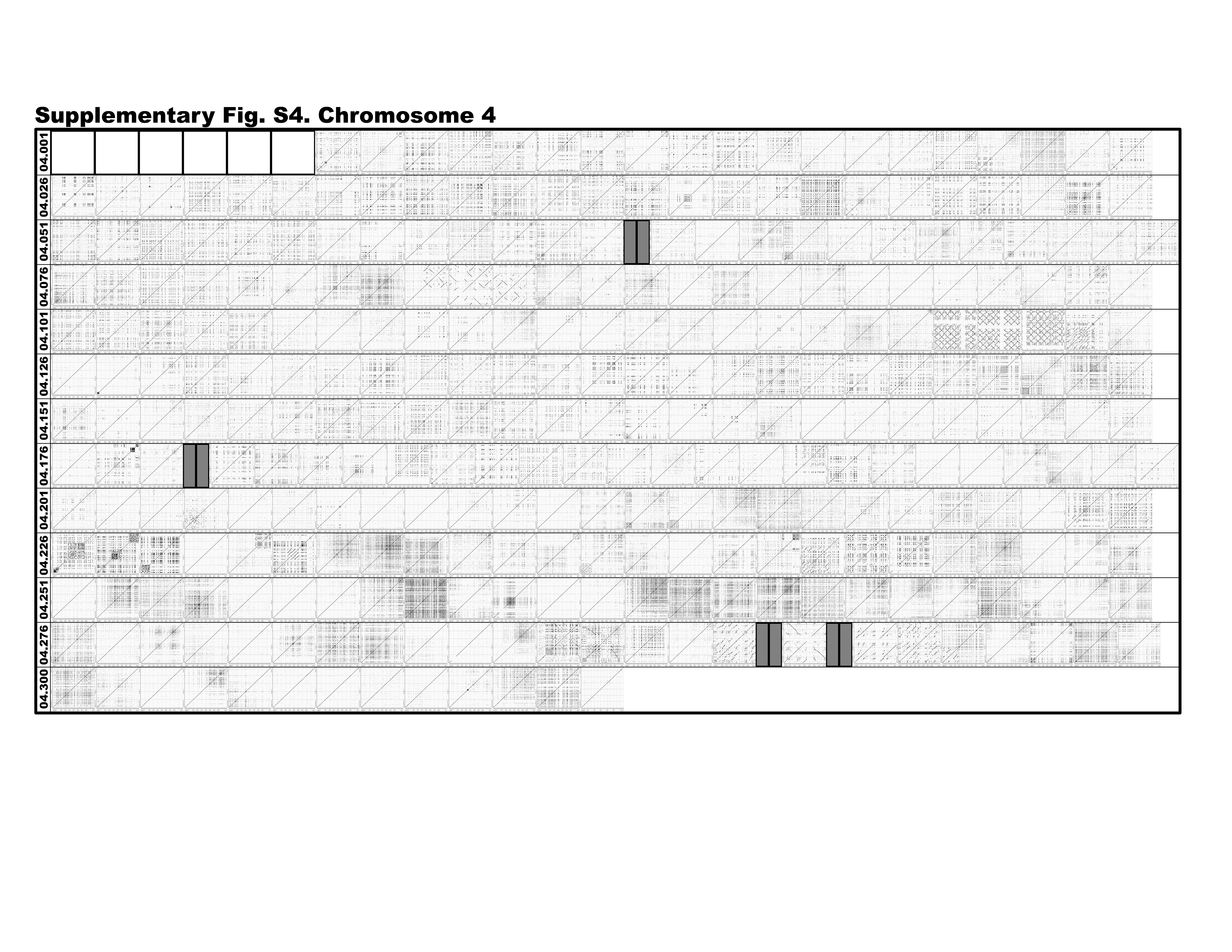

Supplement: Figure S4 — Detailed dot-matrix plot view of the RE arrays in the mouse chromosome 4 from Fig. 2 . The dot-matrix plots of the self-alignment data derived from a total of 312 genome units of 0.5 Mb are compiled for the mouse chromosome 4. Each genome unit is represented by a square and unit identifications are indicated only for the ones on the far left of each row. Genome units without any sequence information (gap) are indicated with a white square. Grey rectangles indicate partial gaps. (TIF) [file pone.0035156.s004.tif]

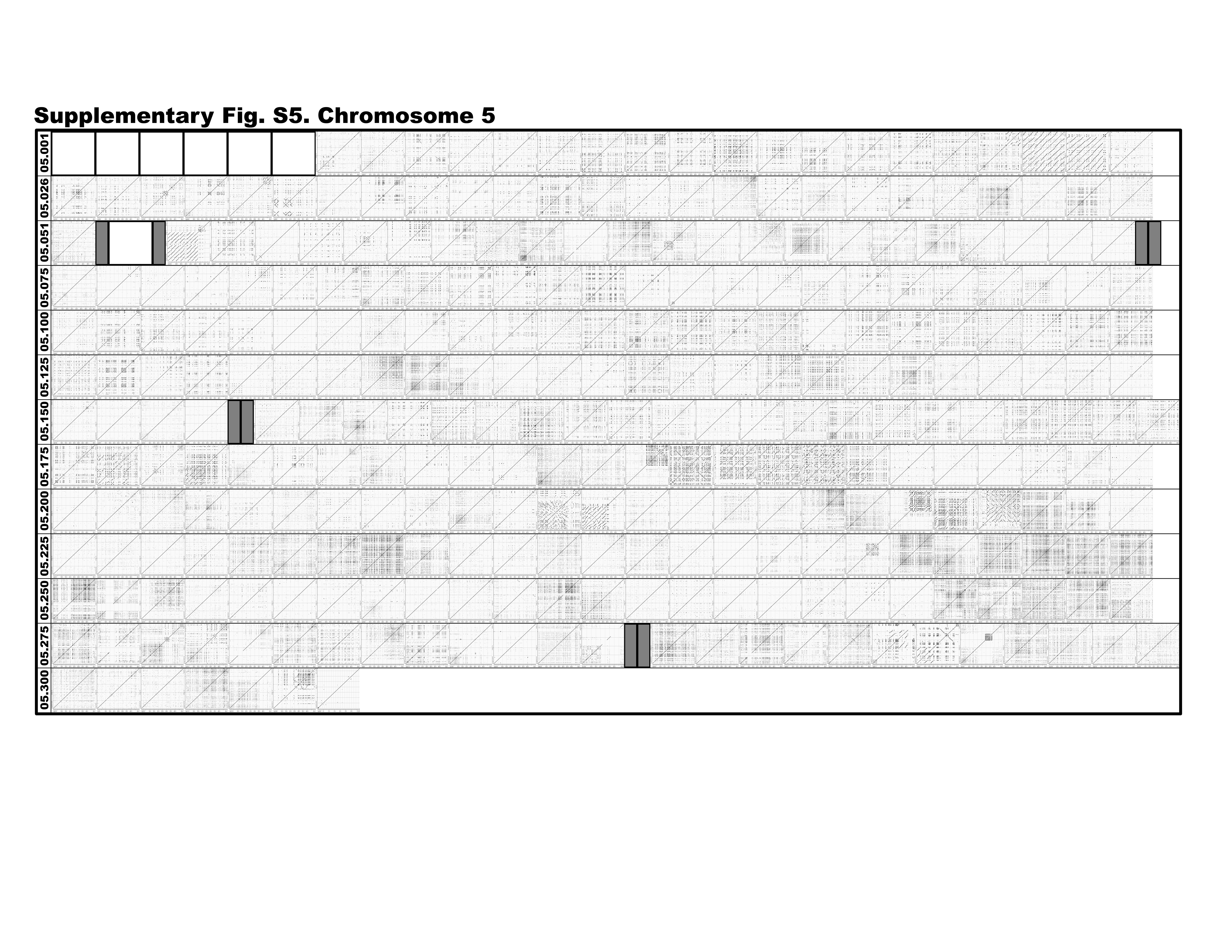

Supplement: Figure S5 — Detailed dot-matrix plot view of the RE arrays in the mouse chromosome 5 from Fig. 2 . The dot-matrix plots of the self-alignment data derived from a total of 306 genome units of 0.5 Mb are compiled for the mouse chromosome 5. Each genome unit is represented by a square and unit identifications are indicated only for the ones on the far left of each row. Genome units without any sequence information (gap) are indicated with a white square. Grey rectangles indicate partial gaps. (TIF) [file pone.0035156.s005.tif]

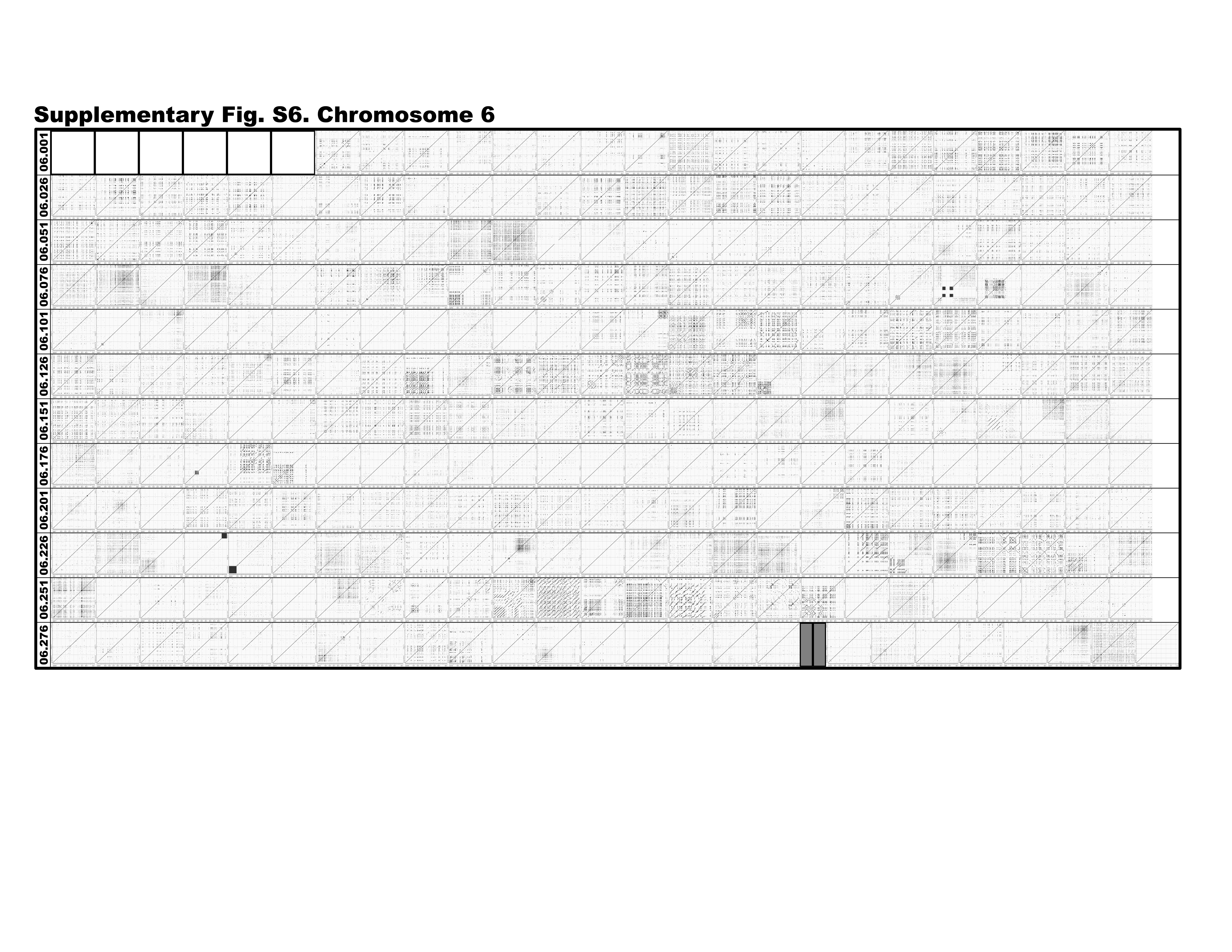

Supplement: Figure S6 — Detailed dot-matrix plot view of the RE arrays in the mouse chromosome 6 from Fig. 2 . The dot-matrix plots of the self-alignment data derived from a total of 300 genome units of 0.5 Mb are compiled for the mouse chromosome 6. Each genome unit is represented by a square and unit identifications are indicated only for the ones on the far left of each row. Genome units without any sequence information (gap) are indicated with a white square. Grey rectangles indicate partial gaps. (TIF) [file pone.0035156.s006.tif]

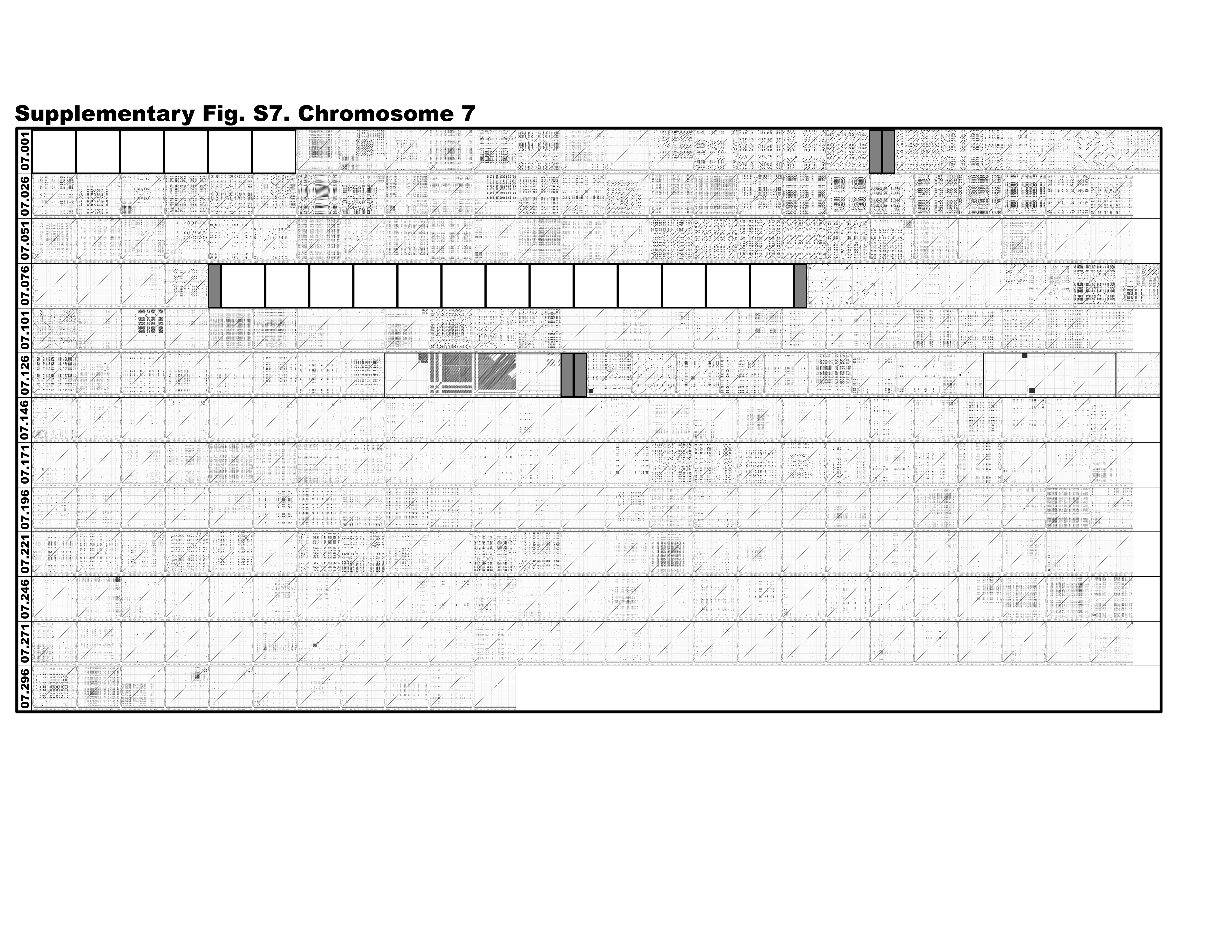

Supplement: Figure S7 — Detailed dot-matrix plot view of the RE arrays in the mouse chromosome 7 from Fig. 2 . The dot-matrix plots of the self-alignment data derived from a total of 306 genome units of 0.5 Mb are compiled for the mouse chromosome 7. Each genome unit/subunit is represented by a square and unit identifications are indicated only for the ones on the far left of each row. Genome units without any sequence information (gap) are indicated with a white square. Grey rectangles indicate partial gaps. A set of subunits derived from one genome unit are grouped with a rectangle. (TIF) [file pone.0035156.s007.tif]

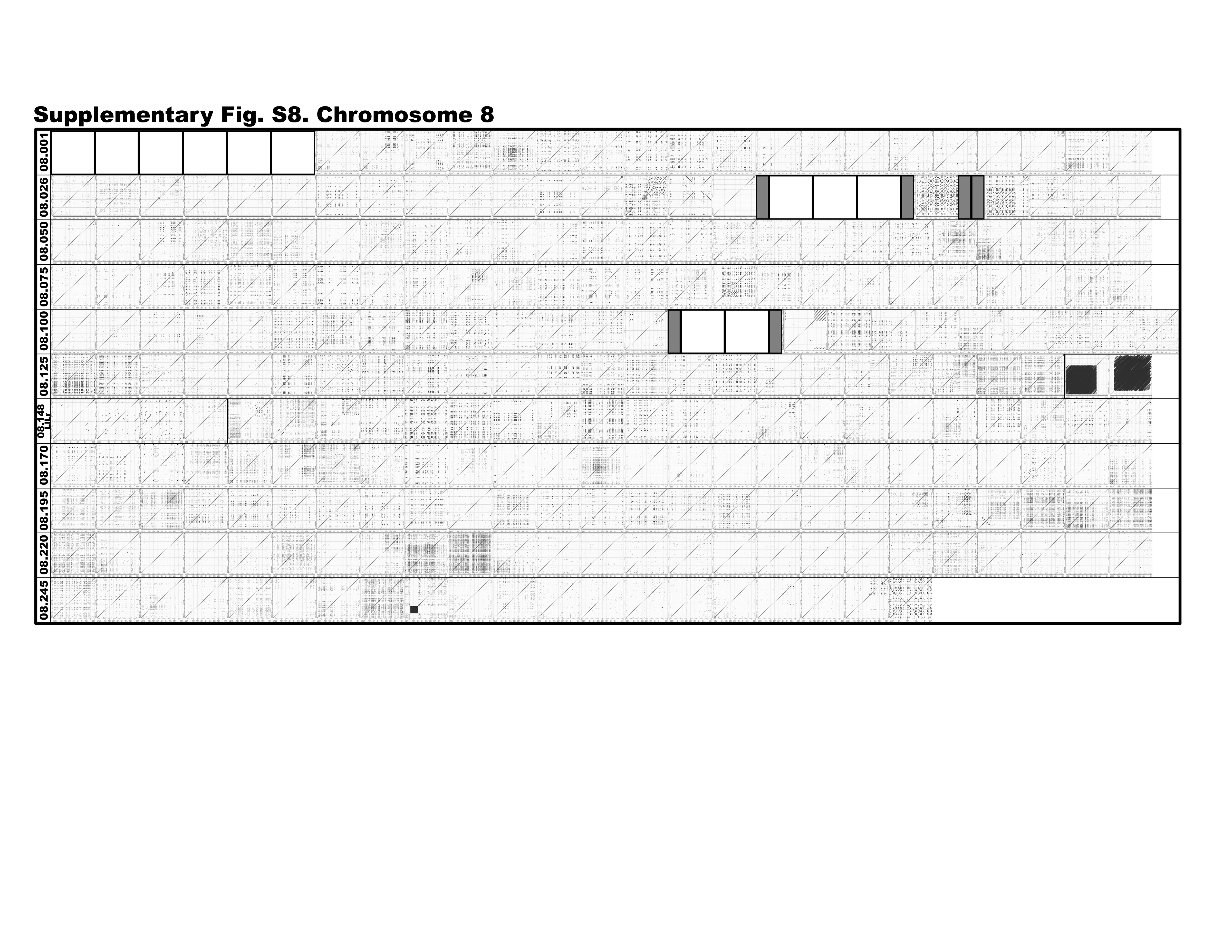

Supplement: Figure S8 — Detailed dot-matrix plot view of the RE arrays in the mouse chromosome 8 from Fig. 2 . The dot-matrix plots of the self-alignment data derived from a total of 264 genome units of 0.5 Mb are compiled for the mouse chromosome 8. Each genome unit/subunit is represented by a square and unit identifications are indicated only for the ones on the far left of each row. Genome units without any sequence information (gap) are indicated with a white square. Grey rectangles indicate partial gaps. A set of subunits derived from one genome unit are grouped with a rectangle. (TIF) [file pone.0035156.s008.tif]

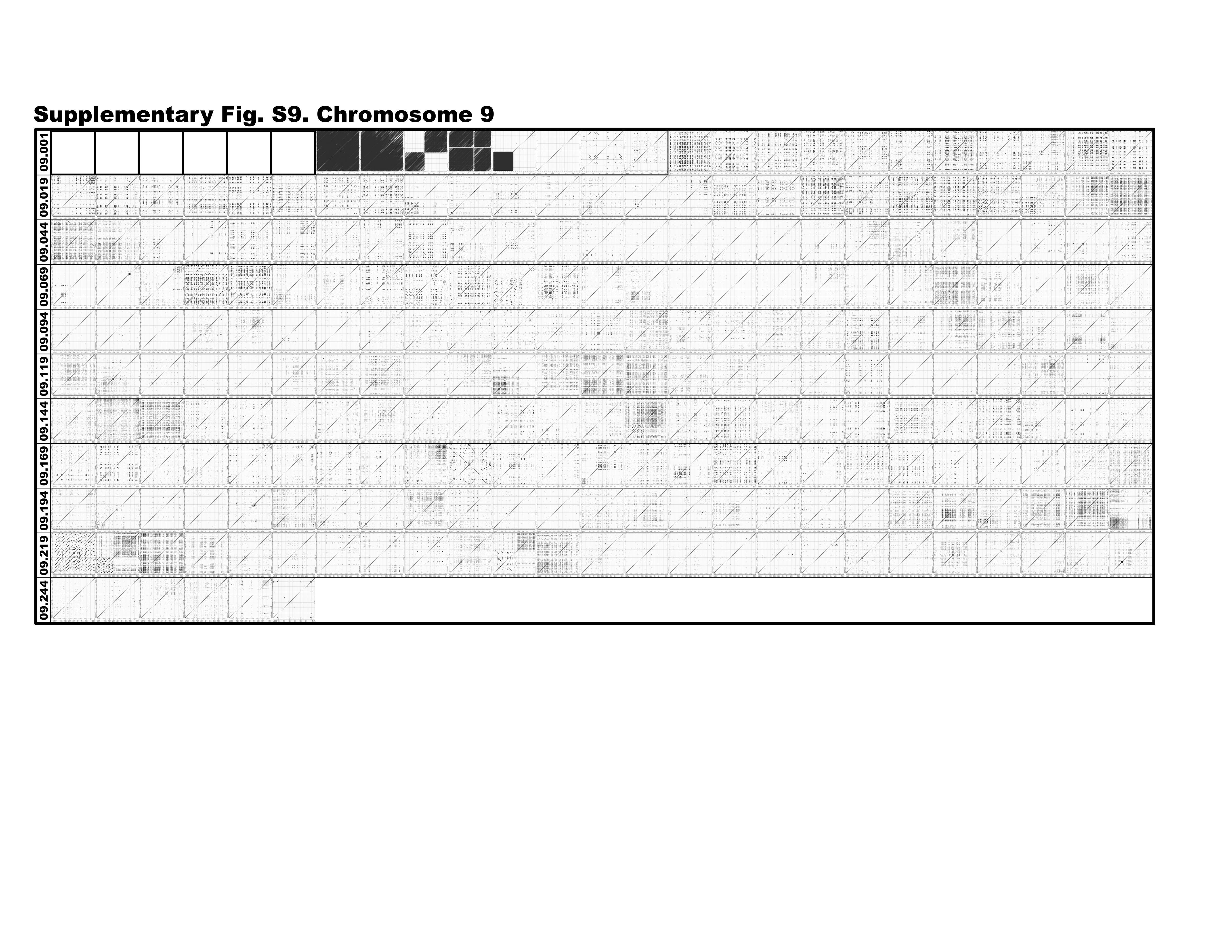

Supplement: Figure S9 — Detailed dot-matrix plot view of the RE arrays in the mouse chromosome 9 from Fig. 2 . The dot-matrix plots of the self-alignment data derived from a total of 249 genome units of 0.5 Mb are compiled for the mouse chromosome 9. Each genome unit/subunit is represented by a square and unit identifications are indicated only for the ones on the far left of each row. Genome units without any sequence information (gap) are indicated with a white square. A set of subunits derived from one genome unit are grouped with a rectangle. (TIF) [file pone.0035156.s009.tif]

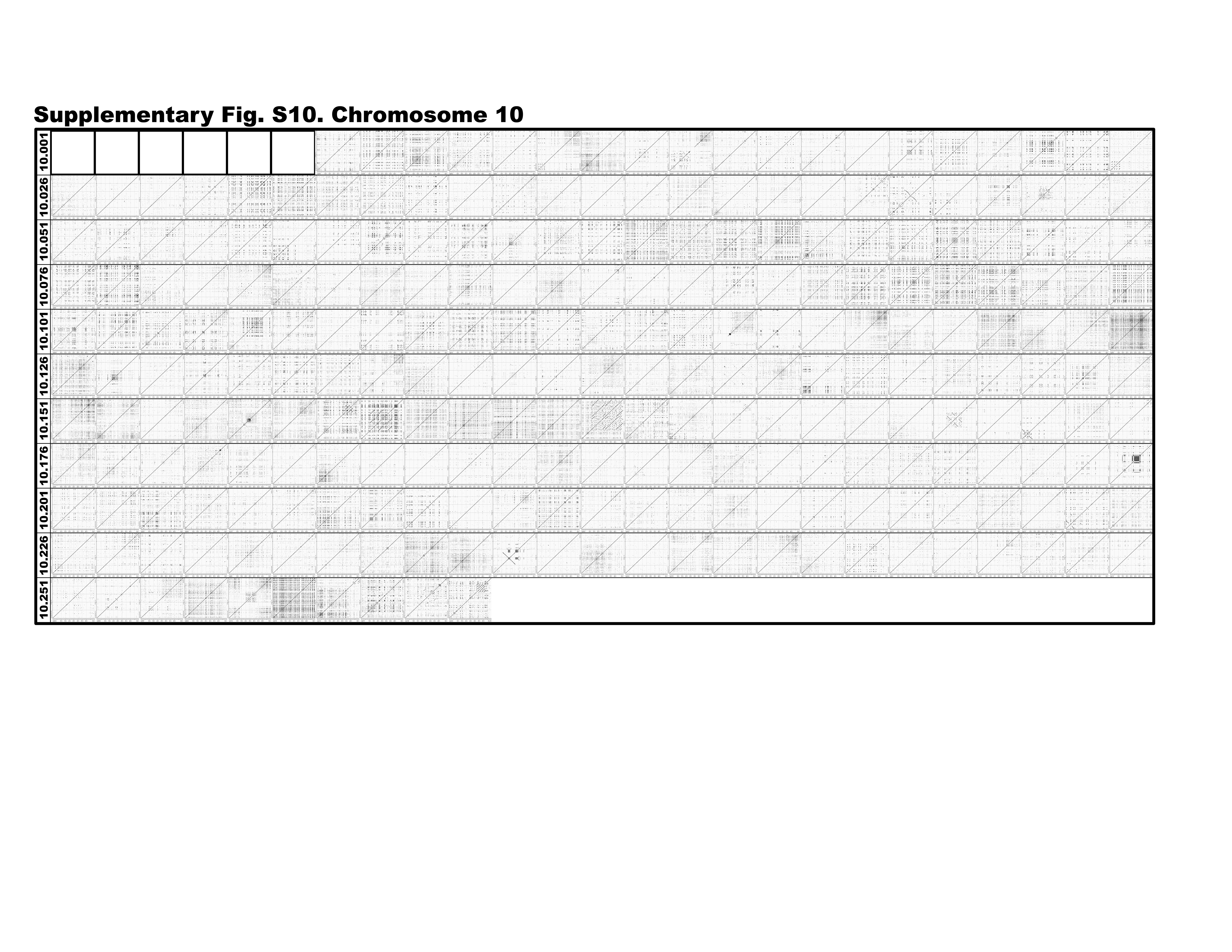

Supplement: Figure S10 — Detailed dot-matrix plot view of the RE arrays in the mouse chromosome 10 from Fig. 2 . The dot-matrix plots of the self-alignment data derived from a total of 260 genome units of 0.5 Mb are compiled for the mouse chromosome 10. Each genome unit is represented by a square and unit identifications are indicated only for the ones on the far left of each row. Genome units without any sequence information (gap) are indicated with a white square. (TIF) [file pone.0035156.s010.tif]

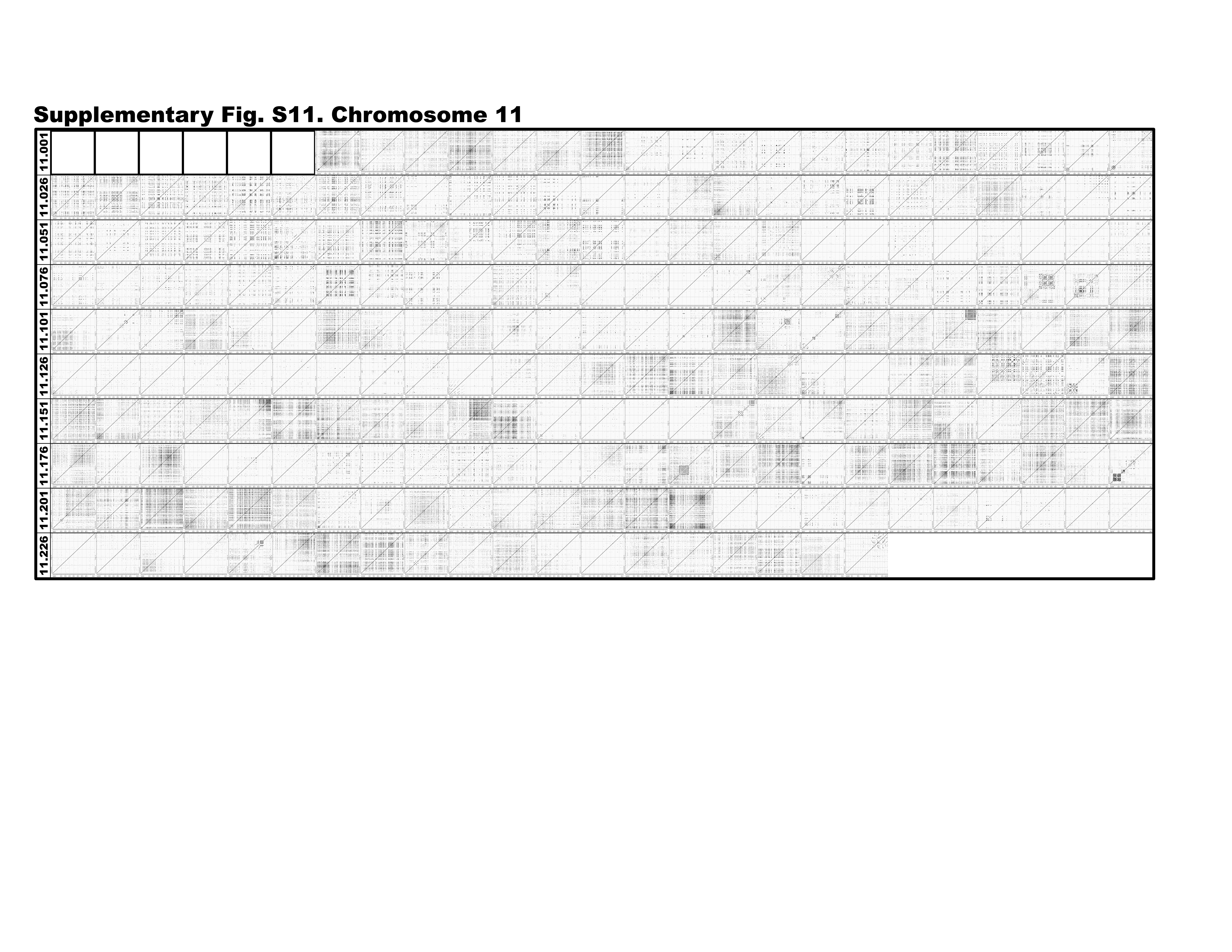

Supplement: Figure S11 — Detailed dot-matrix plot view of the RE arrays in the mouse chromosome 11 from Fig. 2 . The dot-matrix plots of the self-alignment data derived from a total of 244 genome units of 0.5 Mb are compiled for the mouse chromosome 11. Each genome unit is represented by a square and unit identifications are indicated only for the ones on the far left of each row. Genome units without any sequence information (gap) are indicated with a white square. (TIF) [file pone.0035156.s011.tif]

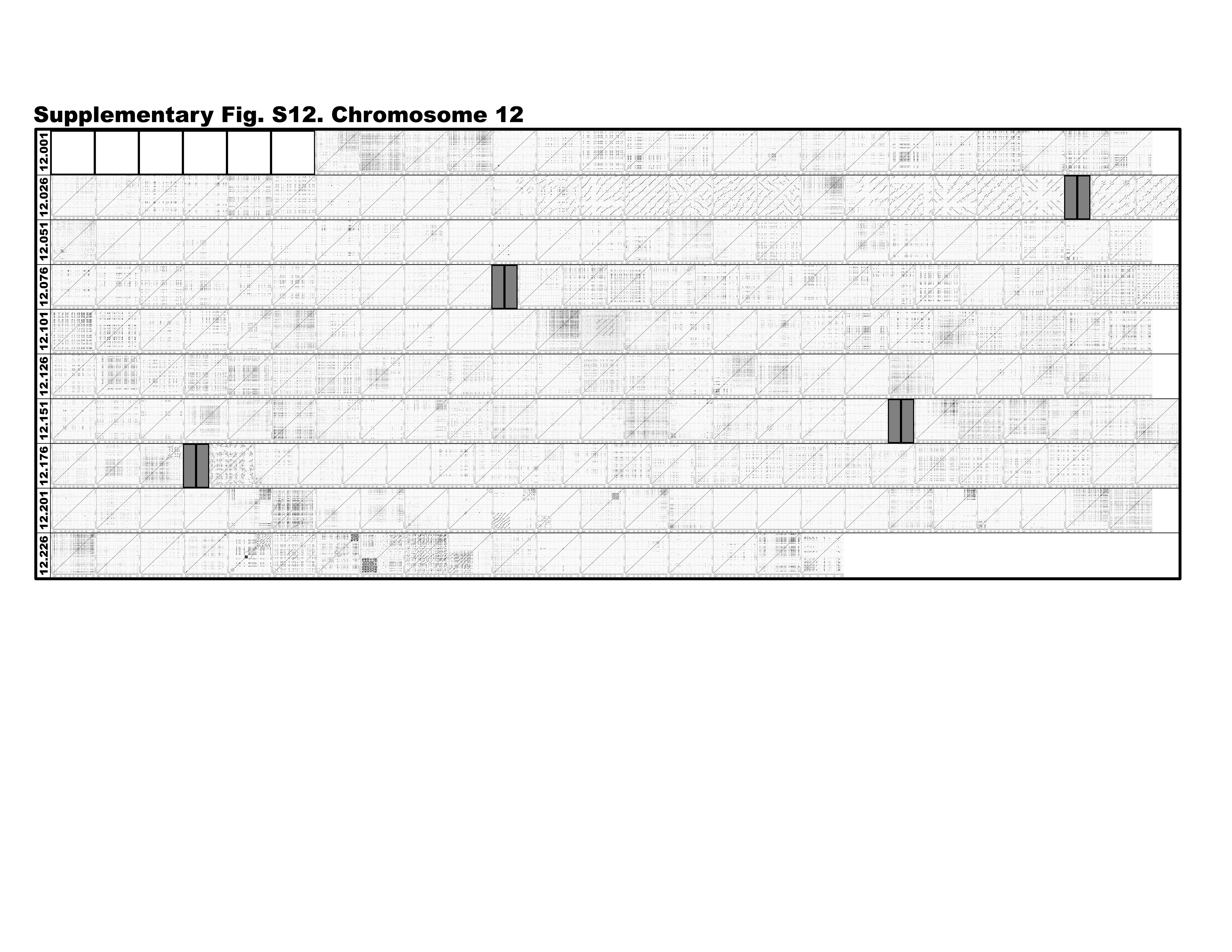

Supplement: Figure S12 — Detailed dot-matrix plot view of the RE arrays in the mouse chromosome 12 from Fig. 2 . The dot-matrix plots of the self-alignment data derived from a total of 243 genome units of 0.5 Mb are compiled for the mouse chromosome 12. Each genome unit is represented by a square and unit identifications are indicated only for the ones on the far left of each row. Genome units without any sequence information (gap) are indicated with a white square. Grey rectangles indicate partial gaps. (TIF) [file pone.0035156.s012.tif]

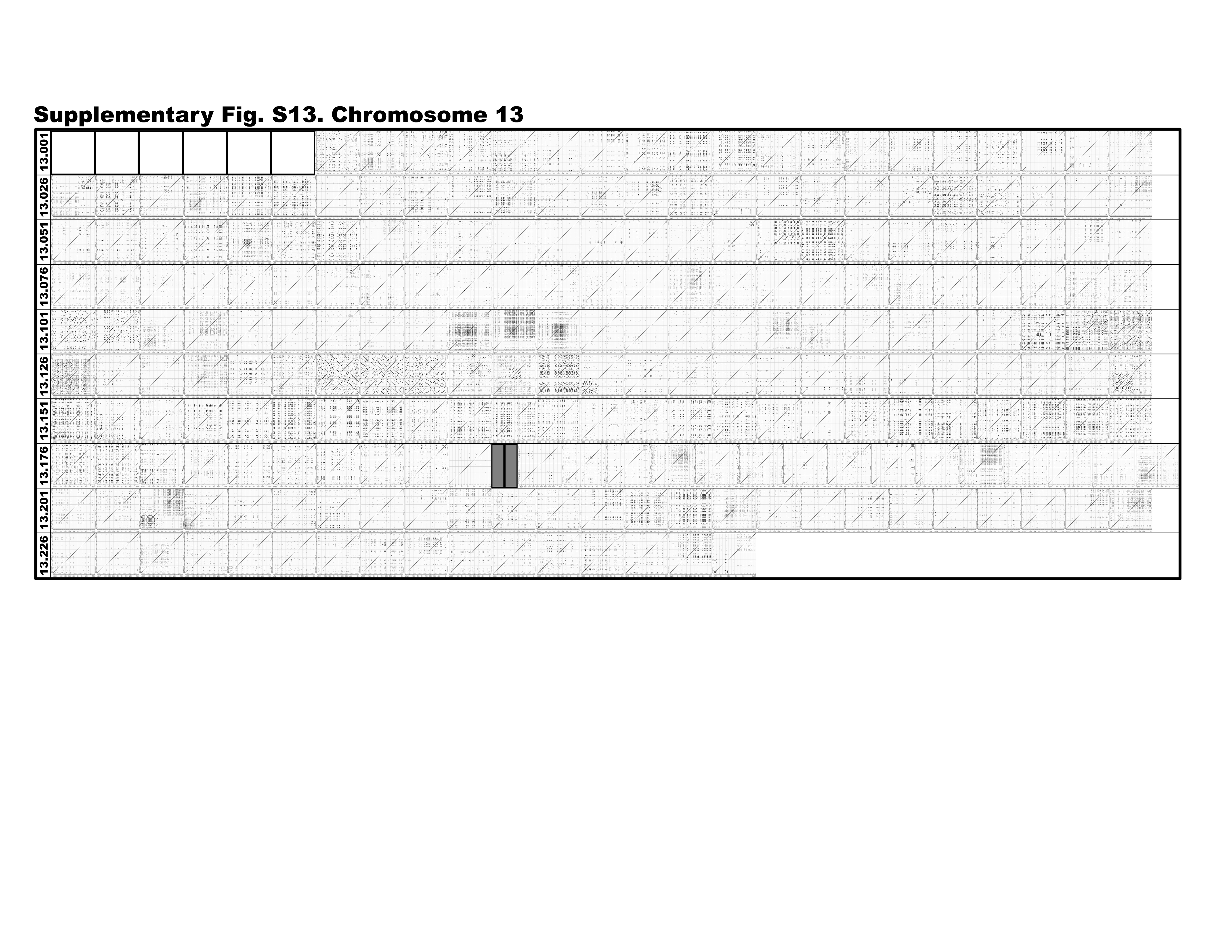

Supplement: Figure S13 — Detailed dot-matrix plot view of the RE arrays in the mouse chromosome 13 from Fig. 2 . The dot-matrix plots of the self-alignment data derived from a total of 241 genome units of 0.5 Mb are compiled for the mouse chromosome 13. Each genome unit is represented by a square and unit identifications are indicated only for the ones on the far left of each row. Genome units without any sequence information (gap) are indicated with a white square. Grey rectangles indicate partial gaps. (TIF) [file pone.0035156.s013.tif]

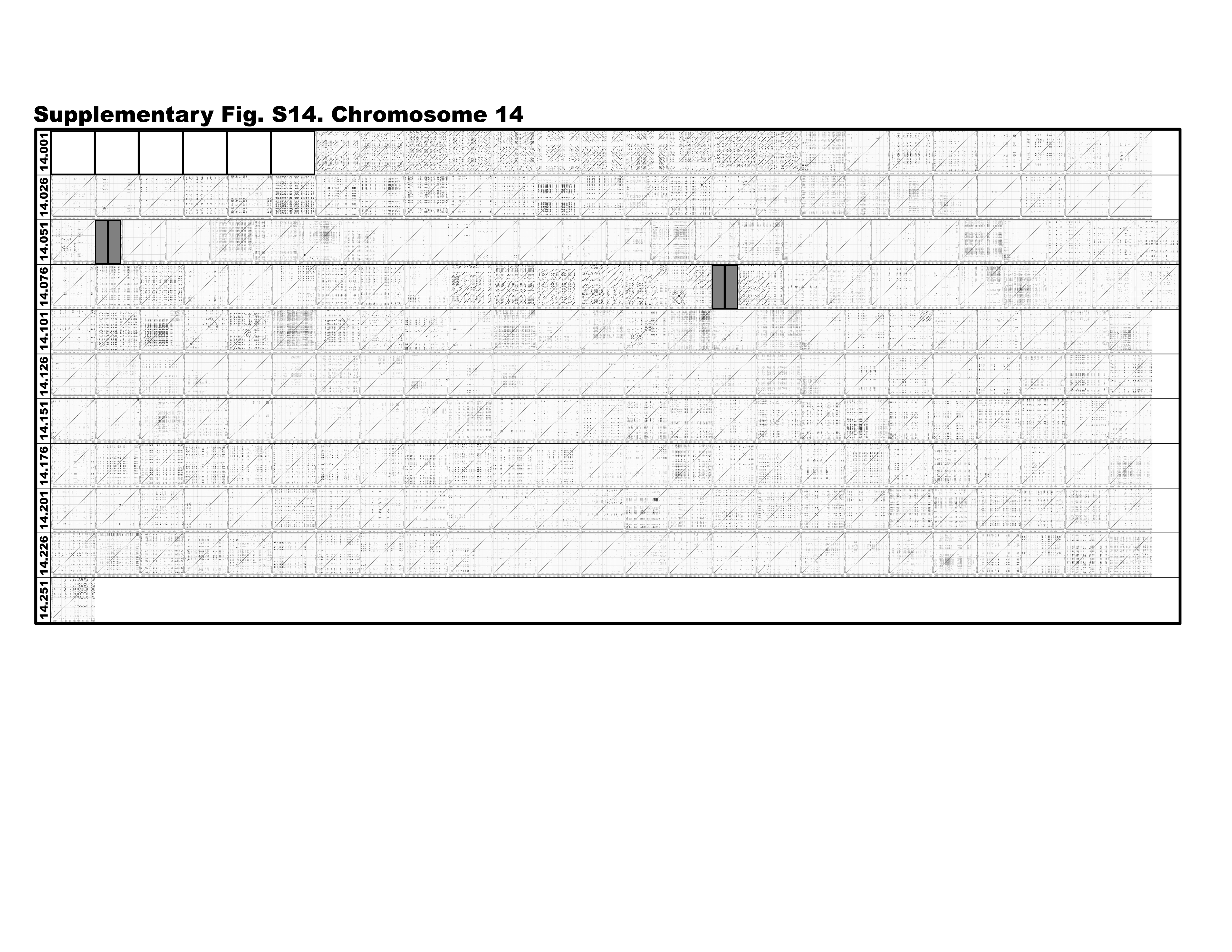

Supplement: Figure S14 — Detailed dot-matrix plot view of the RE arrays in the mouse chromosome 14 from Fig. 2 . The dot-matrix plots of the self-alignment data derived from a total of 251 genome units of 0.5 Mb are compiled for the mouse chromosome 14. Each genome unit is represented by a square and unit identifications are indicated only for the ones on the far left of each row. Genome units without any sequence information (gap) are indicated with a white square. Grey rectangles indicate partial gaps. (TIF) [file pone.0035156.s014.tif]

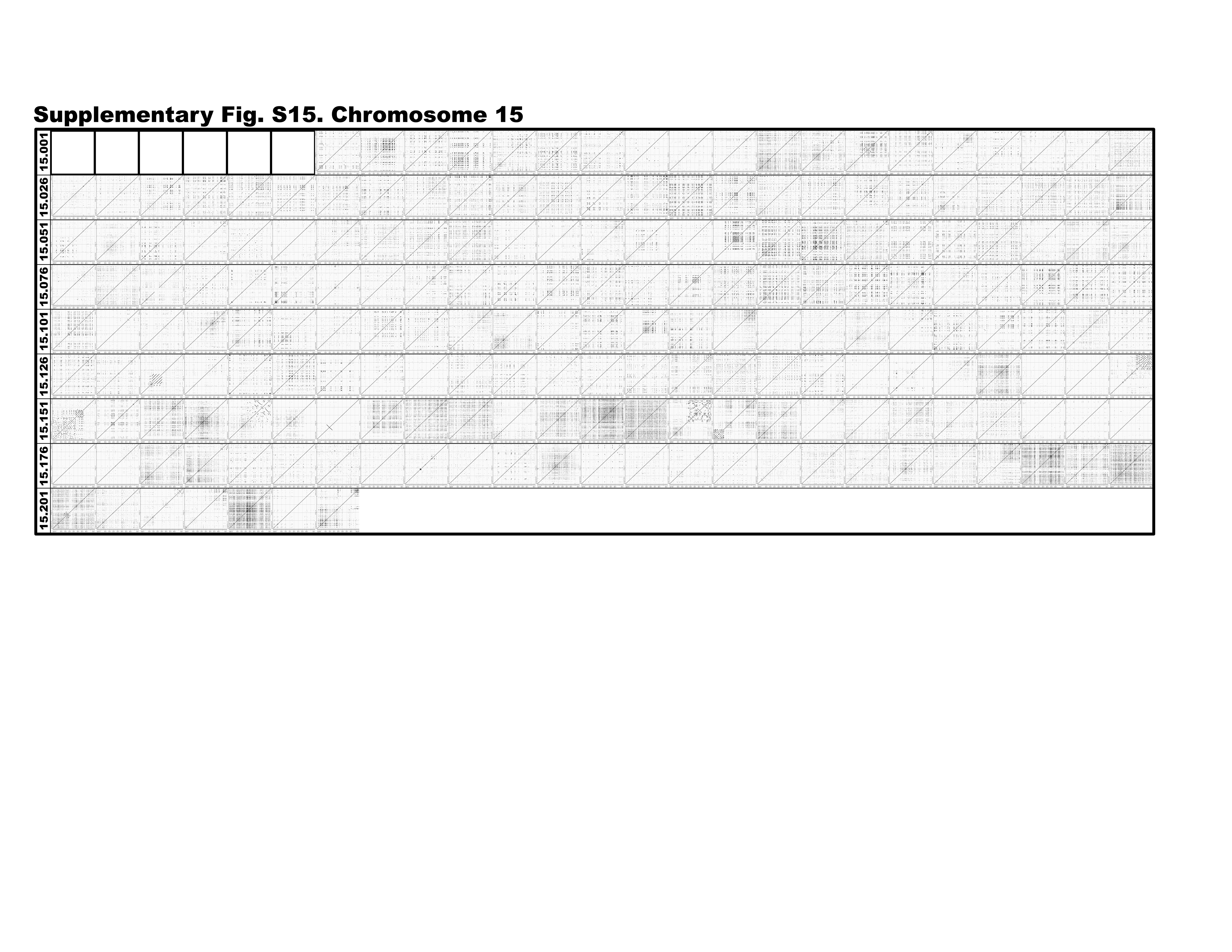

Supplement: Figure S15 — Detailed dot-matrix plot view of the RE arrays in the mouse chromosome 15 from Fig. 2 . The dot-matrix plots of the self-alignment data derived from a total of 207 genome units of 0.5 Mb are compiled for the mouse chromosome 15. Each genome unit/subunit is represented by a square and unit identifications are indicated only for the ones on the far left of each row. Genome units without any sequence information (gap) are indicated with a white square. (TIF) [file pone.0035156.s015.tif]

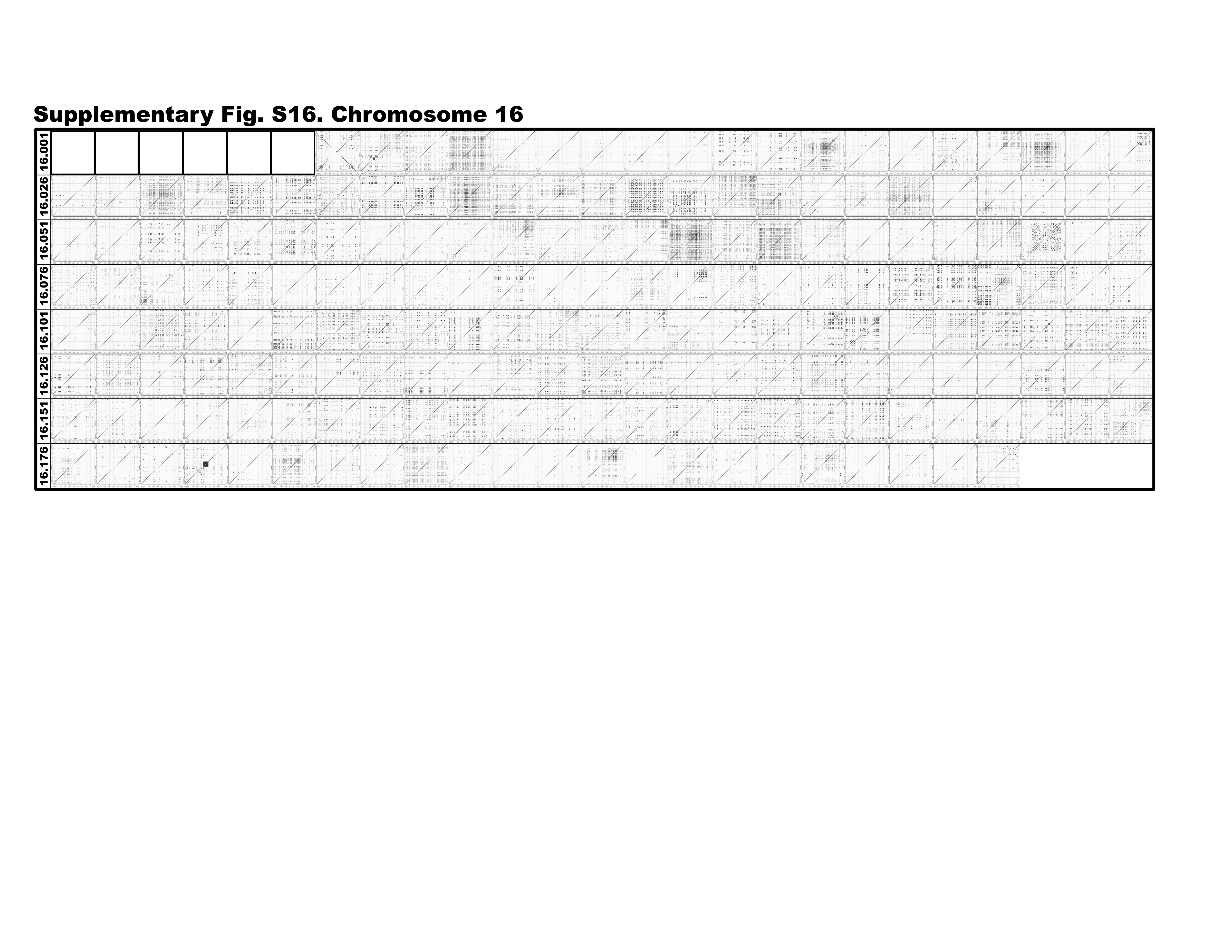

Supplement: Figure S16 — Detailed dot-matrix plot view of the RE arrays in the mouse chromosome 16 from Fig. 2 . The dot-matrix plots of the self-alignment data derived from a total of 197 genome units of 0.5 Mb are compiled for the mouse chromosome 16. Each genome unit is represented by a square and unit identifications are indicated only for the ones on the far left of each row. Genome units without any sequence information (gap) are indicated with a white square. (TIF) [file pone.0035156.s016.tif]

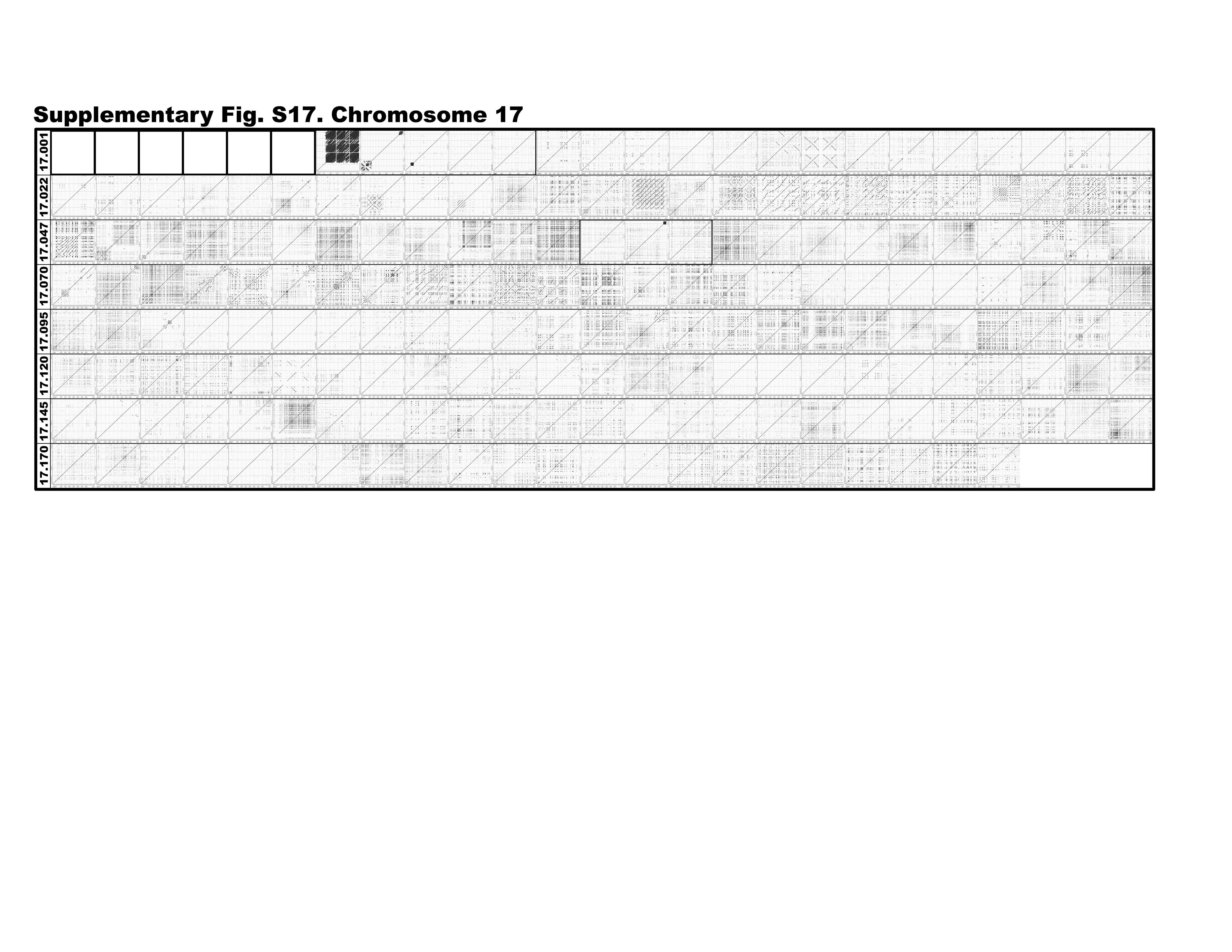

Supplement: Figure S17 — Detailed dot-matrix plot view of the RE arrays in the mouse chromosome 17 from Fig. 2 . The dot-matrix plots of the self-alignment data derived from a total of 191 genome units of 0.5 Mb are compiled for the mouse chromosome 17. Each genome unit/subunit is represented by a square and unit identifications are indicated only for the ones on the far left of each row. Genome units without any sequence information (gap) are indicated with a white square. A set of subunits derived from one genome unit are grouped with a rectangle. (TIF) [file pone.0035156.s017.tif]

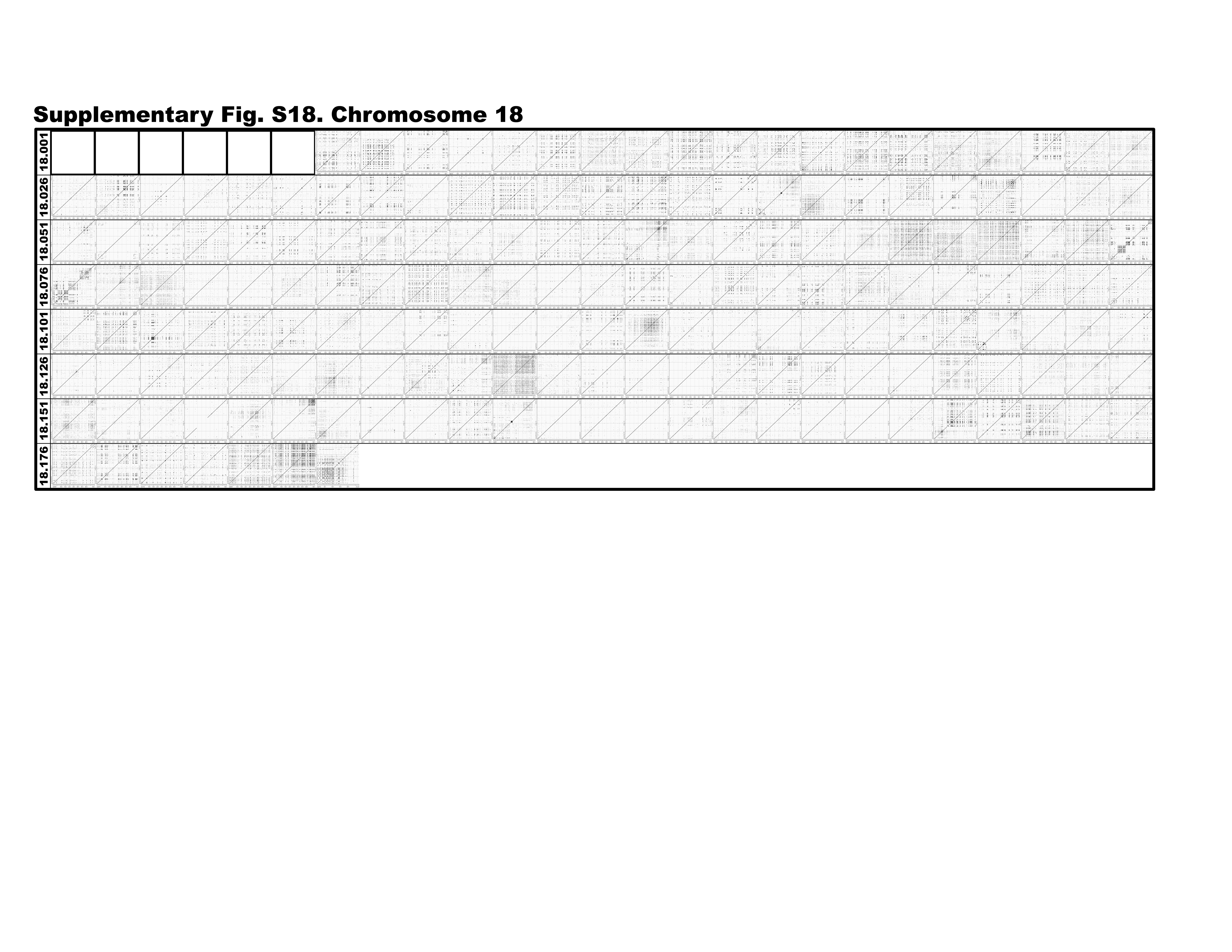

Supplement: Figure S18 — Detailed dot-matrix plot view of the RE arrays in the mouse chromosome 18 from Fig. 2 . The dot-matrix plots of the self-alignment data derived from a total of 182 genome units of 0.5 Mb are compiled for the mouse chromosome 18. Each genome unit is represented by a square and unit identifications are indicated only for the ones on the far left of each row. Genome units without any sequence information (gap) are indicated with a white square. (TIF) [file pone.0035156.s018.tif]

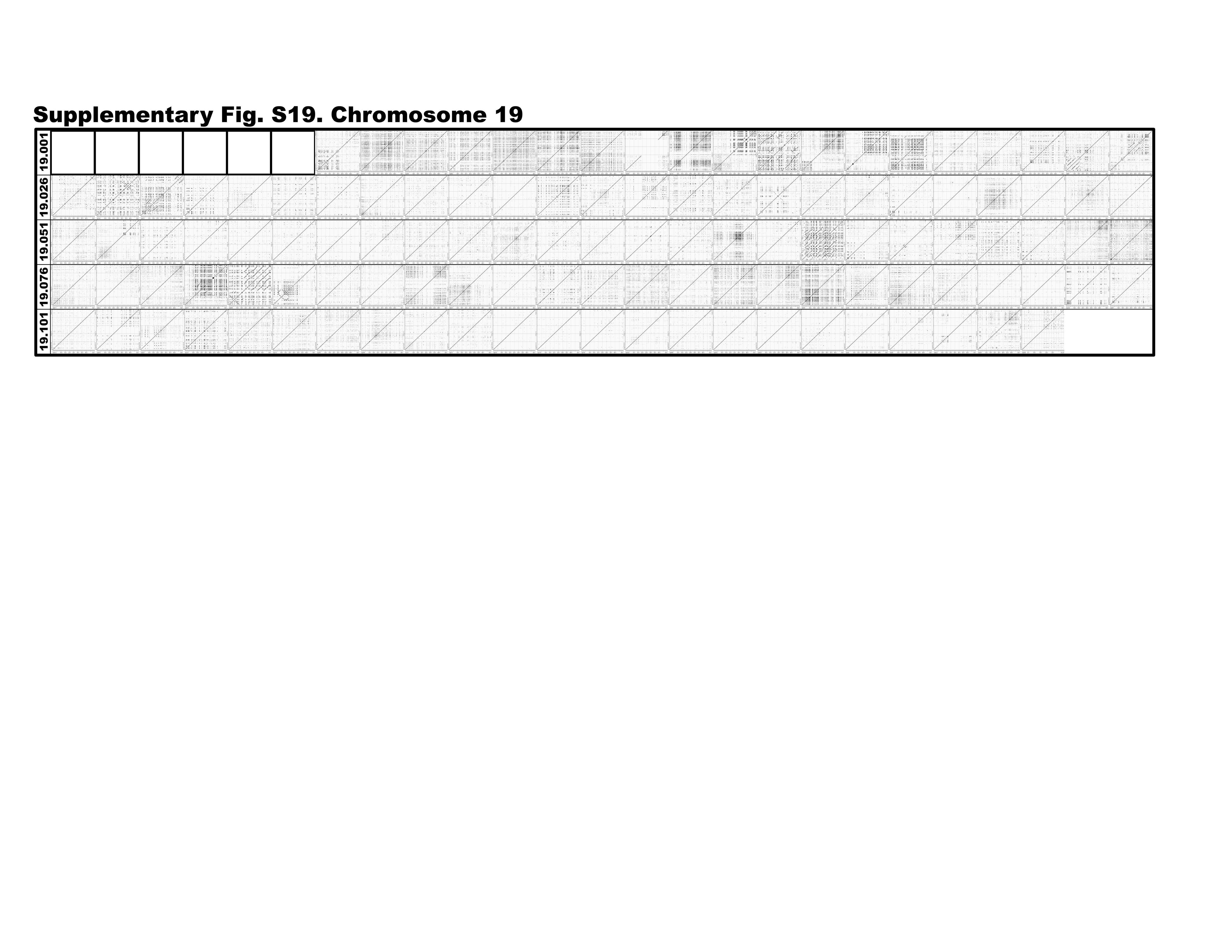

Supplement: Figure S19 — Detailed dot-matrix plot view of the RE arrays in the mouse chromosome 19 from Fig. 2 . The dot-matrix plots of the self-alignment data derived from a total of 123 genome units of 0.5 Mb are compiled for the mouse chromosome 19. Each genome unit is represented by a square and unit identifications are indicated only for the ones on the far left of each row. Genome units without any sequence information (gap) are indicated with a white square. (TIF) [file pone.0035156.s019.tif]

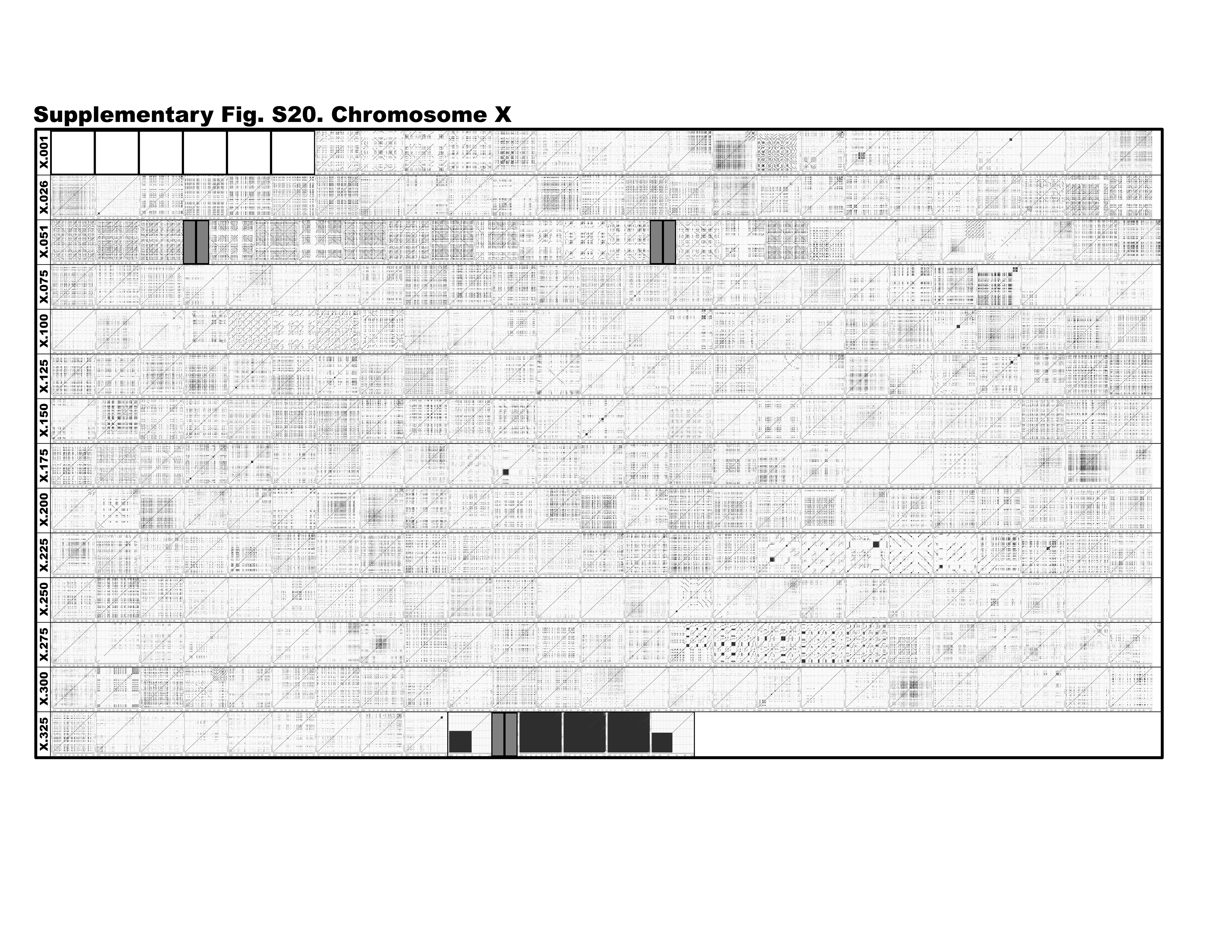

Supplement: Figure S20 — Detailed dot-matrix plot view of the RE arrays in the mouse chromosome X from Fig. 2 . The dot-matrix plots of the self-alignment data derived from a total of 334 genome units of 0.5 Mb are compiled for the mouse chromosome X. Each genome unit/subunit is represented by a square and unit identifications are indicated only for the ones on the far left of each row. Genome units without any sequence information (gap) are indicated with a white square. Grey rectangles indicate partial gaps. A set of subunits derived from one genome unit are grouped with a rectangle. (TIF) [file pone.0035156.s020.tif]

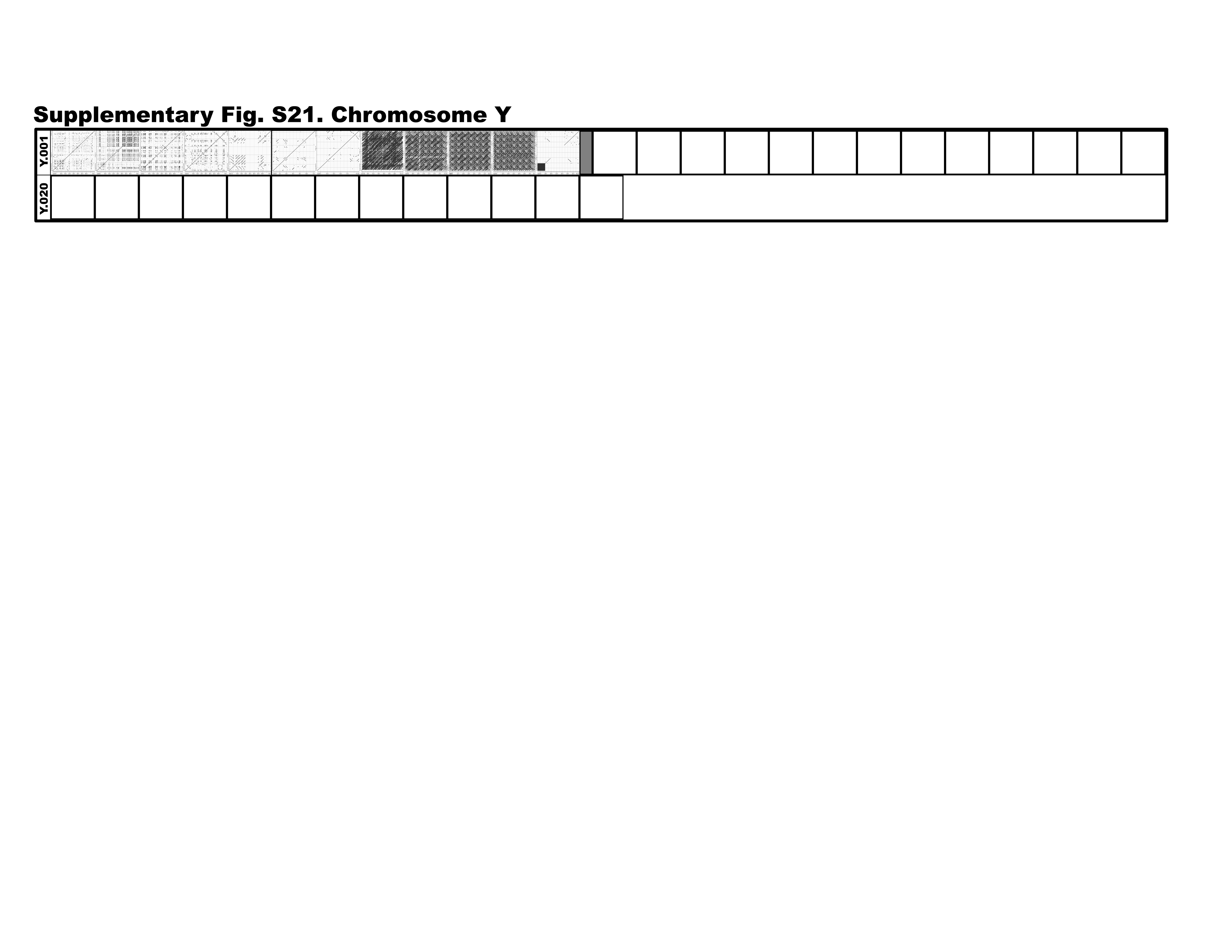

Supplement: Figure S21 — Detailed dot-matrix plot view of the RE arrays in the mouse chromosome Y from Fig. 2 . The dot-matrix plots of the self-alignment data derived from a total of 32 genome units of 0.5 Mb are compiled for the mouse chromosome Y. Each genome unit/subunit is represented by a square and unit identifications are indicated only for the ones on the far left of each row. Genome units without any sequence information (gap) are indicated with a white square. A set of subunits derived from one genome unit are grouped with a rectangle. (TIF) [file pone.0035156.s021.tif]
